# Crosslinking of Linear Polyimines Into Aminal‐Linked Porous Organic Polymers for C_2_ Hydrocarbon/Methane Separation

**DOI:** 10.1002/anie.5692086

**Published:** 2026-06-24

**Authors:** Xuejie Li, Yayu Yan, Qiaohong Li, Michelle Åhlén, Chao Xu

**Affiliations:** ^1^ Institute of Molecular Engineering and Applied Chemistry Anhui University of Technology Ma'anshan P. R. China; ^2^ State Key Laboratory of Structural Chemistry Fujian Institute of Research on the Structure of Matter Chinese Academy of Sciences Fuzhou Fujian P. R. China; ^3^ Division of Nanotechnology and Functional Materials Department of Materials Science and Engineering Ångström Laboratory Uppsala University Uppsala Sweden

**Keywords:** aminal linkages, crosslinking, hydrocarbon separation, porous organic polymers

## Abstract

Hypercrosslinked polymers, a class of porous organic polymers (POPs), are constructed by crosslinking linear polymers or knitting small aromatic molecules with a molecular crosslinker, typically via Friedel–Crafts alkylation. In this study, we report a new approach for the synthesis of POPs via the crosslinking of linear polyimines with *m*‑phenylenediamine through nucleophilic addition of amines to imines, forming aminal linkages. The resulting aminal‐linked POPs, with an estimated low cost of 16 USD/kg, exhibited high surface areas up to 650 m^2^/g and abundant microporosity, in contrast to the ∼100 m^2^/g observed for the linear polyimine. This method demonstrated good generality: four linear polyimines with different structures were successfully crosslinked into POPs with high porosity. Both experimental results and theoretical calculations indicate that the use of diamines at the meta‐position is critical for efficient crosslinking, whereas para‐diamines did not initiate crosslinking. With their high surface area and rich microporosity, these POPs displayed high adsorption capacities for C_2_ hydrocarbons and CO_2_, but significantly lower capacity for CH_4_. Dynamic breakthrough experiments confirmed excellent separation performance for C_2_ hydrocarbons/CH_4_ mixtures, highlighting their potential for hydrocarbon separation. This study provides a new strategy for the synthesis of cost‐effective POPs and demonstrates their promising applications in gas separation.

## Graphical abstract

Crosslinking of linear polyimines with aromatic diamines via nucleophilic addition converts nonporous polyimine precursors into aminal‐linked porous networks, offering a new strategy for synthesizing porous organic polymers. The resulting materials exhibit high C_2_ hydrocarbon adsorption capacity, excellent selectivity over CH_4_, and durable cyclic stability and moisture tolerance, as demonstrated by dynamic breakthrough experiments.

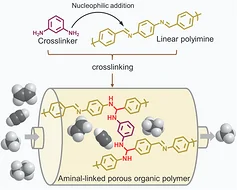

## 1 Introduction

Porous organic polymers (POPs) represent an important class of emerging porous materials [1]. POPs are constructed exclusively from organic building blocks interconnected through strong covalent bonds to form extended polymeric networks [2]. Based on their crystallinity and molecular architectures, POPs can be broadly classified into several subclasses, including covalent organic frameworks (COFs) [3, 4], covalent triazine frameworks (CTFs) [5, 6, 7], conjugated microporous polymers (CMPs) [8, 9], polymers of intrinsic microporosity (PIMs) [10, 11], and hypercrosslinked polymers (HCPs) [12, 13]. Owing to their unique structural characteristics, POPs exhibit high specific surface areas, abundant microporosity, tunable pore sizes, and tailorable surface chemistry, which clearly distinguish them from conventional polymers. As a result, POPs have been extensively investigated for a wide range of applications, including gas storage [14, 15], separation and purification [16, 17, 18, 19, 20, 21], heterogeneous catalysis [22, 23, 24, 25, 26, 27], and energy storage and conversion [28, 29, 30, 31, 32, 33].

Among these subclasses, HCPs represent the earliest example of POPs, first developed in the 1970s by Davankov and co‐workers [34, 35]. They also became the first commercially available POP materials, with common products including hypercrosslinked polystyrene adsorbent resins (e.g., Macronet‐type resins) that have been widely used in adsorption and separation processes [36, 37]. Distinct from other POPs that are typically constructed by covalently linking small organic monomers through coupling or condensation polymerization reactions, HCPs are generally synthesized via Friedel–Crafts alkylation reactions, either by post‐crosslinking pre‐formed polymer chains or by “knitting” small aromatic molecules using external crosslinkers [12, 38, 39, 40, 41, 42, 43, 44, 45]. Compared with other types of POPs, HCPs offer a significant advantage in terms of low production cost, enabling large‐scale synthesis and practical commercialization.

Despite their early commercialization and attractive cost advantages, the structural diversity of HCPs remains largely limited by their synthetic chemistry. To date, the vast majority of HCPs have been prepared via Friedel–Crafts alkylation reactions, which typically require strong Lewis acid catalysts and electron‐rich aromatic substrates [13]. This over‐reliance on a single reaction motif not only raises concerns regarding sustainability and functional‐group tolerance, but also severely constrains the accessible network architectures and chemical functionalities of HCP materials. Developing alternative crosslinking chemistries that enable structurally diverse, heteroatom‐rich, and low‐cost HCPs under mild conditions therefore remains a significant challenge.

Schiff base condensation has been widely used in the synthesis of various POPs, including both amorphous and crystalline materials, the latter also known as COFs [46, 47]. These materials are typically prepared by polycondensation between organic monomers bearing multiple amine and aldehyde groups, leading to the formation of imine linkages as the most common and stable products of the reaction. However, to construct two‐ or three‐dimensional (2D or 3D) polymer networks, which are essential for generating high porosity, at least one monomer must possess three or more functional groups. Such monomers are often expensive, with costs ranging from hundreds to thousands of USD per gram, which significantly increases the production cost and limits large‐scale manufacturing. In addition to imine formation, Schiff base condensation can also generate aminal linkages as transient intermediates [48]. Moreover, imine exchange reactions between amines and imines can also result in aminal formation [49, 50]. The formation of aminal linkages offers opportunities to construct new types of POPs. In particular, the sp^3^‐hybridized carbon center in an aminal linkage can serve as a branching site within the polymer network, facilitating the formation of 2D or 3D architectures and generating porosity when inexpensive diamine and dialdehyde monomers are employed. This feature enables the synthesis of cost‐effective POPs. For example, Laybourn et al. reported the synthesis of POPs with specific surface areas of up to 600 m^2^/g using aromatic diamines and dialdehydes under solvothermal conditions [51]. Recently, we reported a large‐scale, cost‐effective, and green approach for the synthesis of imine‐ and aminal‐linked POPs under mild conditions, with estimated production costs as low as 10 USD/kg [52, 53, 54]. Given the possibility of forming aminal linkages in polymer networks, we envision that crosslinking non‐porous linear polyimines with aromatic diamines via aminal formation could generate high porosity in the resulting polymer networks, thereby enriching the synthetic routes to POPs.

Herein, we report the crosslinking of linear polyimines using *m*‐phenylenediamine (MPA) as a crosslinker to construct POPs through the formation of aminal linkages. The resulting POPs exhibit a significantly enhanced surface area of up to 650 m^2^/g compared to the parent one‐dimensional (1D) polyimines (∼50–100 m^2^/g). The method shows good generality, as four different 1D polyimines were successfully crosslinked into aminal‐linked POPs. The resulting POPs, featuring rich microporosity, display high adsorption capacities toward C_2_ hydrocarbons and good selectivity over CH_4_. The potential of these materials for hydrocarbon separation was further evaluated by dynamic breakthrough experiments, demonstrating high separation efficiency, good moisture resistance, and excellent recyclability.

## 2 Results and Discussion

### 2.1 Synthesis and Characterization of POPs

**FIGURE 1:**
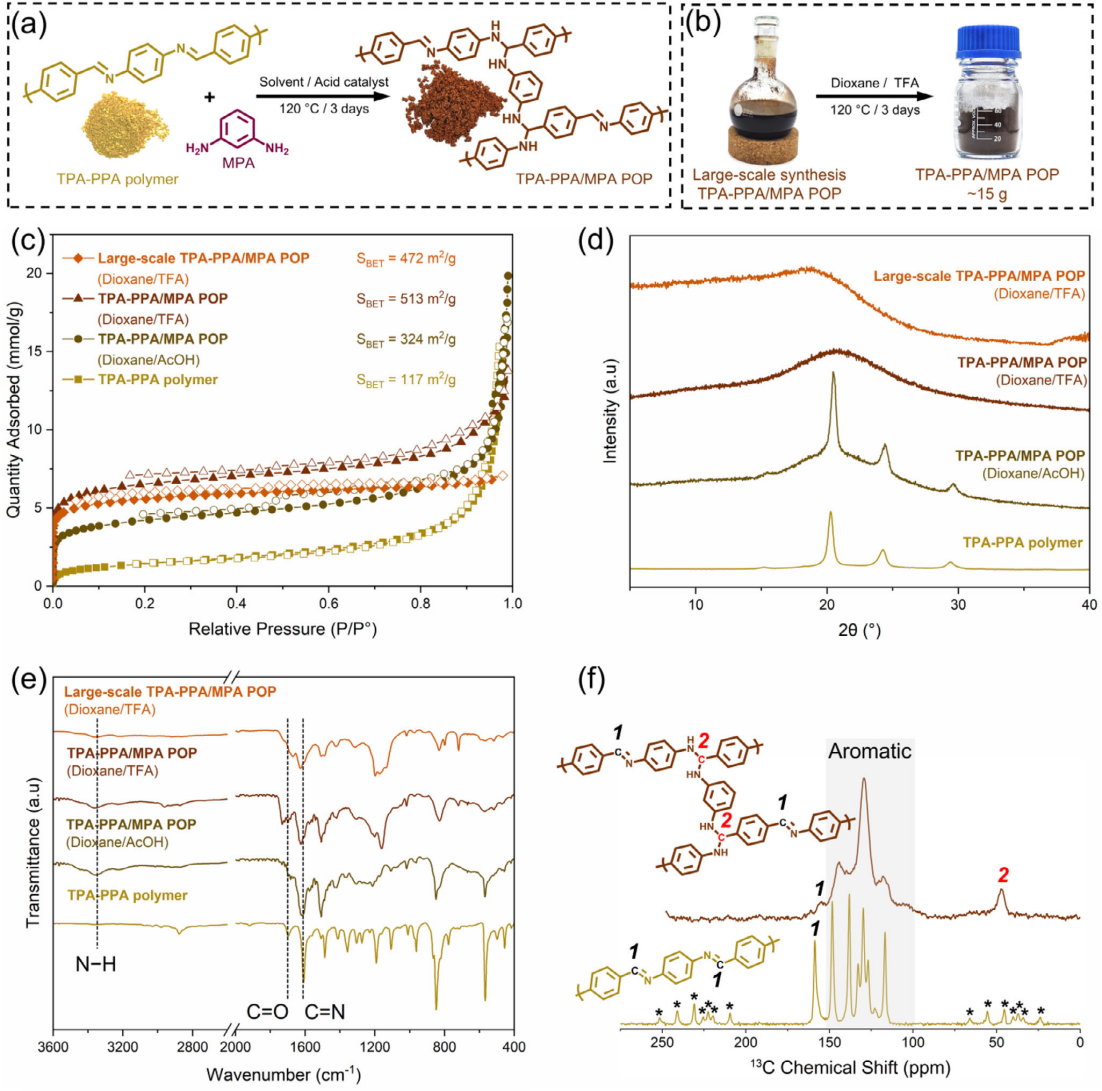
(a) Synthesis route for crosslinking the 1D TPA–PPA polymer with MPA to form aminal‐linked porous organic polymers (POPs). Optical images show the color change from the yellow TPA–PPA precursor to the dark brown POP product. (b) Optical images of a large‐scale synthesis of TPA‐PPA/MPA POP. (c) N_2_ sorption isotherms recorded at 77 K, (d) powder X‐ray diffraction (PXRD) patterns, and (e) infrared (IR) spectra of TPA–PPA polymer and TPA–PPA/MPA POPs. (f) solid‐state ^13^C nuclear magnetic resonance (NMR) spectra of 1D TPA–PPA polymer [52] and TPA–PPA/MPA POP. Asterisks (*) denote side‐spinning sidebands.

The 1D polyimine precursor was prepared by the condensation of terephthalaldehyde (TPA) and *p*‐phenylenediamine (PPA) in ethanol, forming a 1D TPA–PPA polymer in the presence of acetic acid (AcOH) as a catalyst. Following conventional solvothermal conditions for POP synthesis, the TPA–PPA polymer and the crosslinker MPA in a molar ratio of 1:1 were mixed in dioxane with an acid catalyst, and the reaction vessel was sealed and heated at 120°C for 3 days. The resulting products displayed a dark brown color, markedly different from the yellow precursor polymer, indicating the possible formation of a crosslinked structure (Figure 1a). Scaling up the synthesis in a round‐bottom flask under the same conditions furthermore yielded a comparable dark brown product on a multigram scale, highlighting the scalability and robustness of the synthetic approach (Figures 1b–e and S11). When trifluoroacetic acid (TFA) and AcOH were used as catalysts, the resulting materials, denoted as TPA–PPA/MPA POPs, exhibited high Brunauer–Emmett–Teller (BET) specific surface areas of 548 and 346 m^2^/g, respectively, significantly greater than that of the TPA–PPA precursor (116 m^2^/g) (Figure 1c). Pore size distribution analysis indicated the presence of abundant micropores in the POPs, with pore sizes centered at 0.8 nm (Figure S1), likely arising from the highly crosslinked structure. The relatively low surface area of the TPA–PPA polymer is attributed to dense packing of the linear polymer chains and the lack of intrinsic porosity. Powder X‐ray diffraction (PXRD) analysis revealed that the TPA–PPA polymer is highly crystalline, exhibiting sharp diffraction peaks at 2θ = 20.3°, 24.3°, and 29.4°, consistent with an ordered arrangement of the 1D polymer chains [55]. In contrast, the crosslinked product obtained using TFA displayed a predominantly amorphous structure, as the characteristic diffraction peaks of the precursor disappeared after the reaction (Figure 1d). Meanwhile, the product obtained using AcOH retained diffraction peaks corresponding to the TPA/PPA polymer, albeit with decreased intensity, indicating incomplete conversion of the crystalline 1D polymer into a POP structure. These observations are consistent with its lower surface area compared to the material obtained using TFA, further demonstrating that TFA is a more efficient catalyst for promoting the crosslinking reaction.

The molecular structures of the TPA–PPA polymer and the resulting POPs were investigated using infrared (IR) spectroscopy, solid‐state ^13^C nuclear magnetic resonance (NMR) spectroscopy, and X‐ray photoelectron spectroscopy (XPS). The TPA–PPA polymer exhibited a strong C═N stretching vibration at 1610 cm^−1^ and no significant N─H stretching bands, indicating that most amine groups from PPA had been consumed during imine formation (Figures 1e and S2a). While a substantial C═O stretching band at 1697 cm^−1^ can be observed in the IR spectrum, corresponding to the terminal end‐group on the polymer chains. In contrast, the TPA–PPA/MPA POPs displayed a pronounced band at 3366 cm^−1^ corresponding to N─H stretching, suggesting that additional N─H bonds were formed through the conversion of imine linkages into aminal linkages during crosslinking. Meanwhile, the POPs still displayed a C═N stretching band at 1625 cm^−1^, although with decreased intensity and a slight shift to higher wavenumbers. This observation indicates that C═N bonds are partially retained in the POPs, implying that only a fraction of the imine bonds participated as reaction sites for crosslinking with MPA to form aminal linkages. The slight blue shift of the C═N vibration may be attributed to partial protonation of the imine groups under acidic reaction conditions. The findings from IR spectroscopy were further supported by analysis of the solid‐state ^13^C NMR spectrum. The TPA–PPA polymer precursor showed a strong resonance at around 159 ppm, corresponding to the imine carbon atoms. In contrast, the TPA–PPA/MPA POP exhibited a significantly decreased intensity of the imine carbon signal, while a new peak appeared at around 47 ppm, characteristic of aminal carbon atoms (Figure 1f) [48, 51, 52, 53]. High‐resolution C 1s XPS spectrum of the TPA–PPA/MPA POP, furthermore, displayed a distinct peak at 288.8 eV, which was absent in the imine‐linked TPA–PPA polymer and can be attributed to carbon atoms in the aminal (N─C─N) linkages (Figure S3a). Additionally, the N 1s spectrum could be deconvoluted into two peaks at 399.0 and 400.5 eV, corresponding to imine and aminal nitrogen species, respectively (Figure S3b). The structural differences between the TPA–PPA polymer and TPA–PPA/MPA POP were reflected in the materials’ thermal stability, with the POP decomposing at a slightly lower temperature (400°C) compared to the polymer precursor (505°C) under an N_2_ atmosphere (Figure S4). This difference may in part be attributed to the extended conjugated structure of the 1D TPA–PPA polymer, which is interrupted by the introduction of sp^3^‐hybridized carbons in the aminal linkages, therefore impacting the thermal stability of the POP. These observations collectively provide strong evidence for the successful formation of aminal linkages through the crosslinking of the polyimine chains with MPA molecules, resulting in a POP containing both imine and aminal linkages.

**FIGURE 2:**
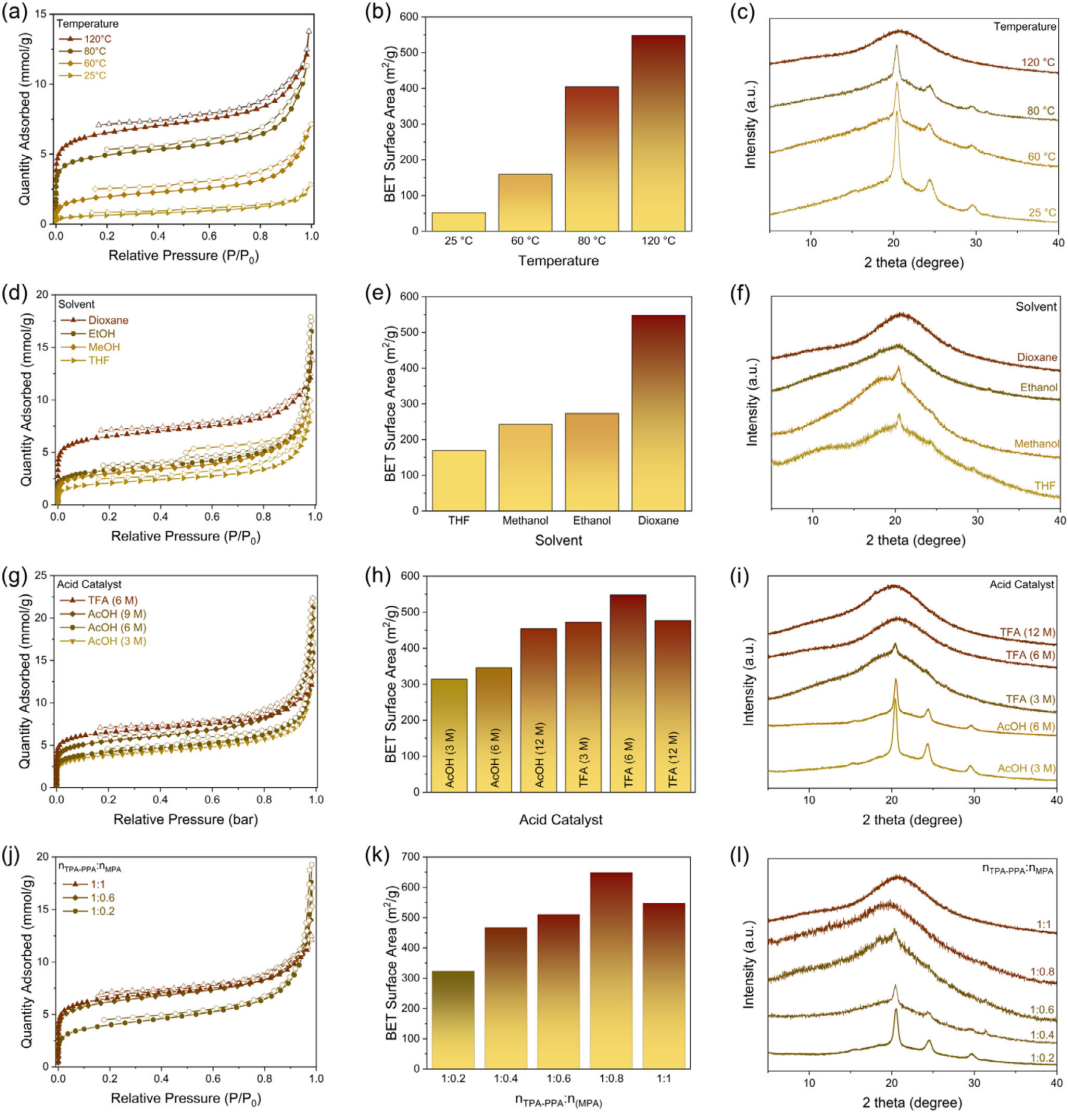
N_2_ sorption isotherms recorded at –196°C (77 K), BET specfic surface areas, and PXRD patterns of TPA–PPA/MPA POPs synthesized under different conditions. (a–c) Different synthesis temperatures (catalyst: 6 M trifluoroacetic acid (TFA); solvent: dioxane). (d–f) Different solvents (catalyst: 6 M TFA; temperature: 120°C). (g–i) Different acid catalysts and concentrations (solvent: dioxane; temperature: 120°C). (j–l) Different molar ratios of TPA–PPA polymer to MPA (catalyst: 6 M TFA; solvent: dioxane; temperature: 120°C). The molar ratio between TPA–PPA polymer and MPA was 1:1 for samples (a–i). All reactions were conducted for 3 days.

Based on the initial synthesis results, the reaction conditions for preparing TPA–PPA/MPA POPs were systematically optimized to maximize both porosity and crosslinking efficiency. The effects of key reaction parameters—including temperature, solvent, acid type and concentration, as well as the structure and molar ratio of the crosslinker—on the degree of structural conversion and the resulting surface area were comprehensively investigated. Firstly, the reaction temperature proved critical for enabling the crosslinking process. Elevated temperatures of 80°C and 120°C produced highly porous networks with BET specific surface areas of 405 and 548 m^2^/g, respectively, indicating the formation of extensively crosslinked structures (Figures 2a,b and S5). In contrast, reactions carried out at lower temperatures of 60°C and 25°C yielded materials with significantly lower surface areas of 160 and 52 m^2^/g, respectively, suggesting insufficient crosslinking. PXRD analysis consistently confirmed that reactions performed at lower temperatures resulted in residual 1D polyimine precursors (Figure 2c). These observations indicate that the imine‐to‐aminal transformation involved in the crosslinking process possesses a higher activation energy barrier than the direct condensation between amine and aldehyde monomers. Secondly, the choice of solvent strongly influenced the extent of crosslinking. Under identical conditions (6 M TFA, 120°C), dioxane yielded a fully amorphous POP with the highest surface area (548 m^2^/g), whereas ethanol, methanol, and THF produced materials with significantly lower surface areas (169–273 m^2^/g) and retained partial crystallinity of the 1D polymer precursor, indicating incomplete structural conversion (Figures 2d–f and S6). Thirdly, the acid catalyst and its concentration also affected the crosslinking efficiency. TFA consistently produced higher surface areas than acetic acid, and increasing the acid concentration improved the porosity of the resulting POPs, with 6 M TFA giving the highest surface area (Figures 2g–i, S7, and S8). Finally, the structure of the crosslinker and the molar ratio between the 1D polyimine and the crosslinker were found to play a critical role in determining both the porosity and the degree of structural conversion. The use of MPA with amine groups in the meta‐position was essential for effective crosslinking, whereas *p*‐phenylenediamine, with amine groups in the para‐position, did not lead to the crosslinking reaction under the same conditions (Figure S9). In addition, increasing the molar ratio of 1D polyimine to MPA from 1:0.2 to 1:0.8 progressively increased the surface area from 324 to 650 m^2^/g, reflecting a higher degree of crosslinking (Figures 2j,k and S10). A further increase of the ratio to 1:1 resulted in a slight decrease in surface area to 548 m^2^/g. Consistently, higher crosslinker ratios produced fully amorphous POPs, while lower ratios retained residual crystalline polymer, indicating that insufficient crosslinker limits complete network formation (Figure 2l). Collectively, these results demonstrate that highly porous and fully crosslinked POPs can be obtained through careful optimization of the reaction temperature, solvent, acid catalyst, and crosslinker.

**FIGURE 3:**
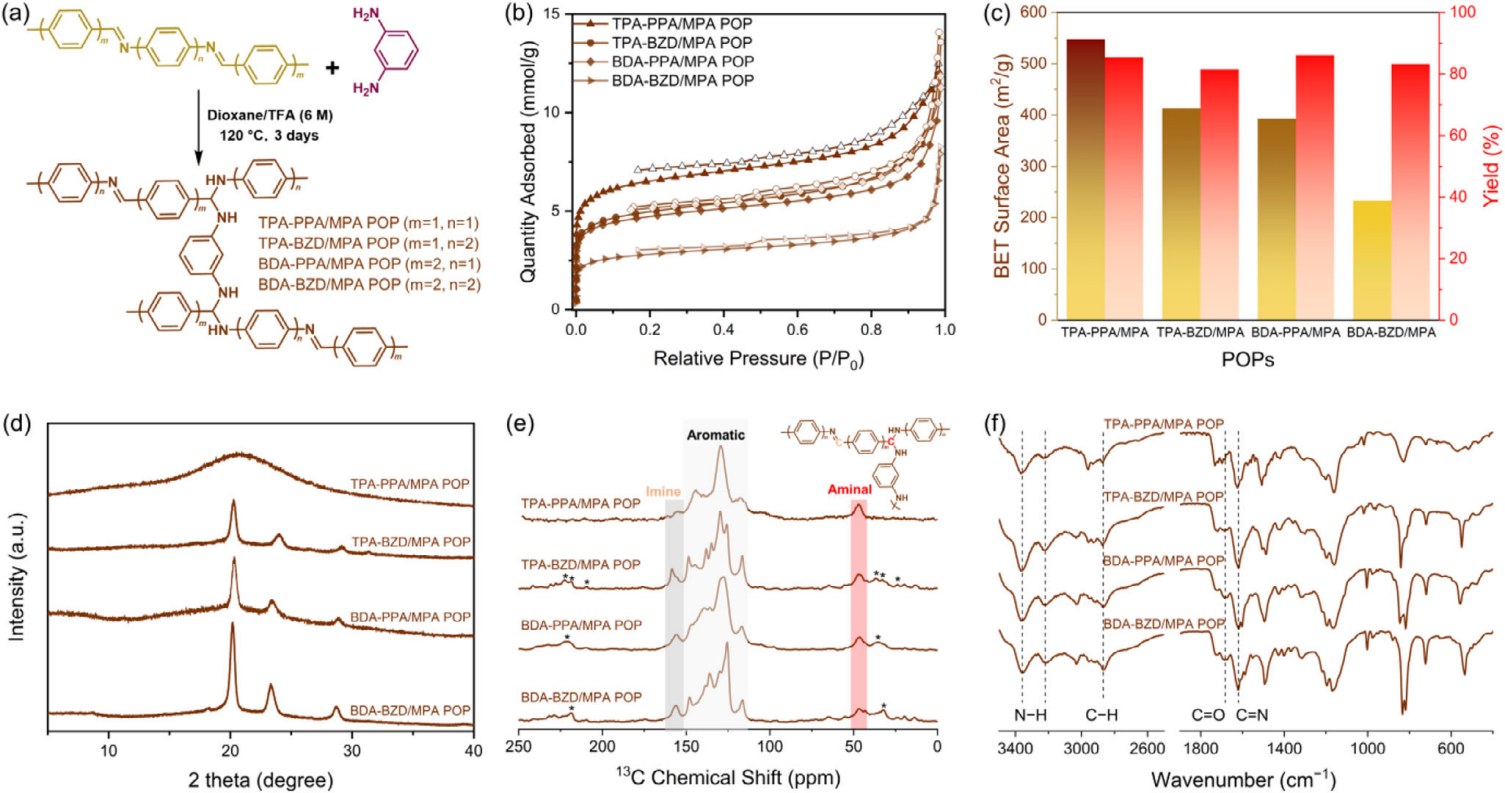
(a) Schematic illustration of the crosslinking of different 1D polyimine precursors with MPA to form aminal‐linked POPs. (b) N_2_ sorption isotherms, (c) BET specific surface areas and yields, (d) PXRD patterns, (e) solid‐state ^13^C NMR spectra, and (f) infrared spectra of POPs synthesized from different polyimine precursors (the intensity of the 3500–2500 cm^−^^1^ region is enlarged to enhance the visibility of the absorption bands). Asterisks (*) denote side‐spinning sidebands.

To further demonstrate the generality of this synthesis strategy for converting 1D polyimines into aminal‐crosslinked POPs, additional polyimine precursors were prepared using aromatic diamine and dialdehyde monomers with different aromatic linker lengths. Specifically, TPA, *p*‐phenylenediamine (PPA), 4,4'‐biphenyldicarboxaldehyde (BDA), and benzidine (BZD) were employed to synthesize a series of 1D polyimine precursors. By combining these diamines and dialdehyde monomers, three additional 1D polyimines—TPA–BZD, BDA–PPA, and BDA–BZD—were obtained (Figure S12a). IR spectroscopy confirmed the formation of polyimine structures through the characteristic imine stretching vibration (Figure S2). All of these polyimines displayed high crystallinity and relatively low BET specific surface areas (50–116 m^2^/g), which can be attributed to the ordered packing of the linear polymer chains and the absence of intrinsic porosity. Upon reaction of these 1D polyimine precursors with MPA under optimized conditions, aminal‐crosslinked POPs were obtained, exhibiting significantly increased surface areas in the range of 233–548 m^2^/g (Figure 3b,c), substantially higher than those of the corresponding polyimine precursors. All reactions gave relatively high yields of >80%. Interestingly, a clear trend was observed: as the length of the aromatic diamine and dialdehyde monomers for the 1D polyimine precursors increased, the resulting crosslinked POPs exhibited progressively lower surface areas. All POPs displayed a nanoflake morphology similar to that of their 1D polyimine precursors (Figure S13). PXRD analysis revealed incomplete conversion of the polyimines derived from monomers with extended aromatic backbones (e.g., TPA–BZD, BDA–PPA, and BDA–BZD) into aminal‐linked POPs. This was evidenced by residual diffraction peaks from unreacted crystalline polyimines, although with significantly reduced intensity compared to the precursors (Figure S14); in contrast, the TPA–PPA precursor exhibited complete conversion into a fully amorphous POP (Figure 3d). This behavior can be attributed to the reduced density of imine sites in polyimines constructed from longer monomers. The increased spacing between neighboring imine groups decreases the probability of reaction with the crosslinker, resulting in a lower crosslinking density and consequently reduced porosity in the resulting POPs. Importantly, studies using solid‐state ^13^C NMR and IR spectroscopies confirmed the formation of aminal bonds in all resulting POPs. The ^13^C NMR spectra exhibited a characteristic signal for aminal carbon atoms at ∼47 ppm, while signals corresponding to imine carbons at ∼155–158 ppm were still observed, indicating the presence of residual imine bonds in the POPs (Figure 3e). Consistently, all POPs showed increased infrared absorption for N─H stretching vibrations around 3360 cm^−1^ compared to their 1D polyimine precursor counterparts, further supporting the formation of aminal linkages during the crosslinking reaction (Figure 3f). The spectra also displayed the C═N stretching vibration at around 1618 cm^−1^, although with decreased intensity relative to the polyimine precursors. The thermal stability of all POPs was lower than that of the corresponding 1D polyimine, which can be attributed to the presence of aminal linkages (Figure S15). Together, these results confirm the successful conversion of 1D polyimines into aminal‐crosslinked POPs, demonstrating the broad applicability and versatility of this synthesis approach.

Notably, all starting materials used in the synthesis of the POPs—including monomers, solvents, and catalysts—are cost‐effective and readily available in large quantities at low industrial prices. As a result, the POPs can be synthesized at very low cost. The estimated production cost of the TPA–PPA/MPA POP is as low as 16 USD/kg at an industrial scale (Table S2, Note: considering solvent and catalyst recycling and accounting only for chemical costs), which is significantly lower than that of most reported porous organic materials and comparable to commercially available sorbents such as activated carbon and zeolites [56, 57]. These findings highlight the strong potential of the POPs for large‐scale industrial synthesis and practical applications.

### 2.2 Mechanism and Theoretical Calculations

**FIGURE 4:**
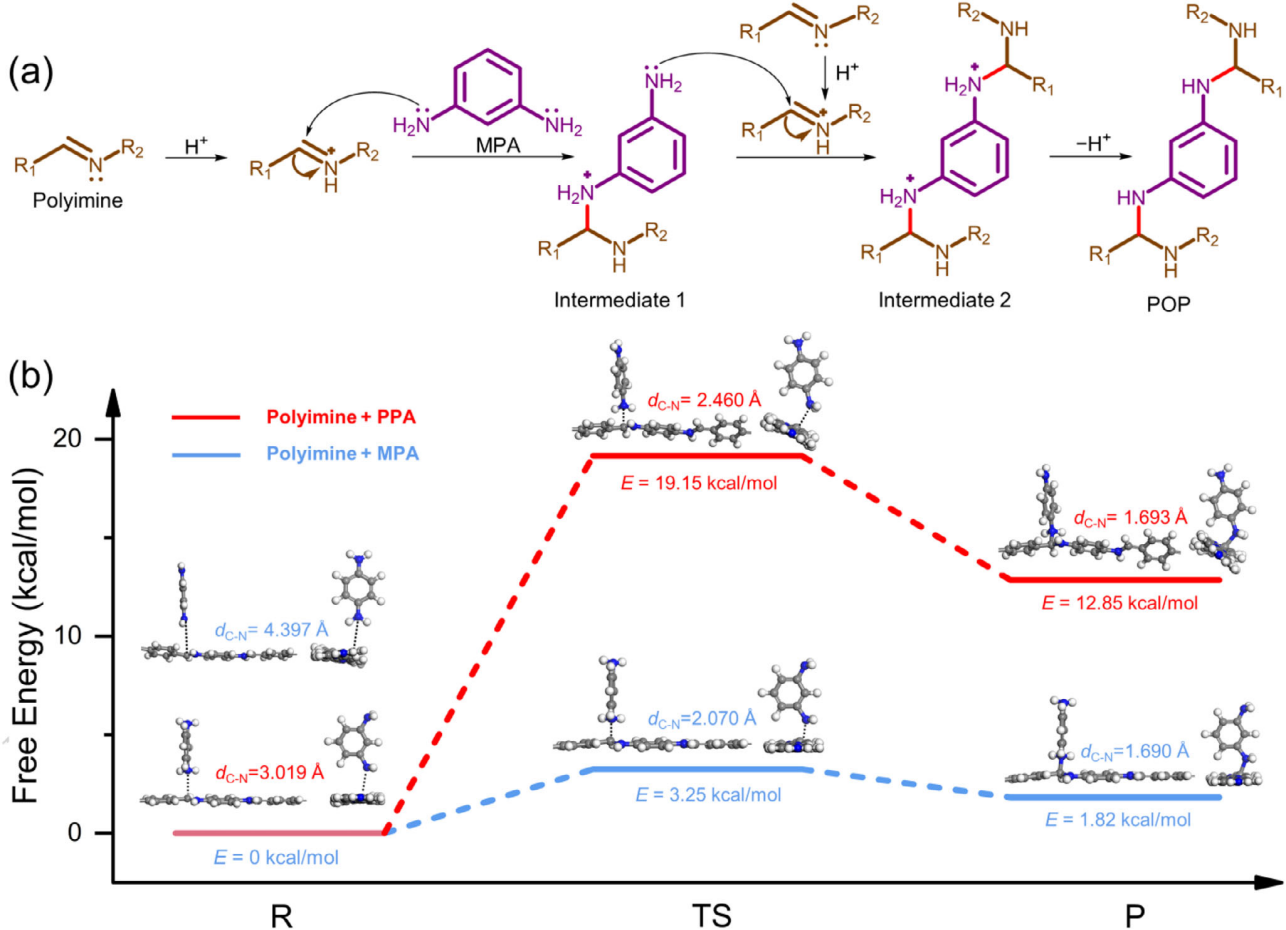
(a) Proposed mechanism for the crosslinking of polyimine with *m*‐phenylenediamine (MPA) to form aminal‐linked POPs. (b) DFT‐calculated reaction free energy for the nucleophilic addition of aromatic diamines, MPA and *p‐*phenylenediamine (PPA), to a protonated imine moiety in a model polyimine fragment. The calculated activation free‐energy barriers and reaction free energies are indicated. Optimized structures of the reactant (R), transition state (TS), and product (P, intermediate 1) are shown from top and side views, with the C─N distances between the nucleophilic amine nitrogen and the imine carbon highlighted.

Based on the experimental results, we propose a mechanism for the transformation of 1D polyimines into aminal‐crosslinked POPs (Figure 4a). In the presence of an acid catalyst, the nitrogen atom of the imine bond is protonated, increasing the electrophilicity of the carbon atom in the C═N bond and making it more susceptible to nucleophilic attack. One amine group of the MPA crosslinker then attacks the electrophilic carbon of the protonated polyimine, forming a protonated intermediate 1. The remaining amine group of MPA can subsequently react with another protonated polyimine chain to generate crosslinked intermediate 2. Finally, deprotonation of intermediate 2 yields the neutral aminal‐crosslinked POP product.

To quantitatively evaluate the energetics and feasibility of this crosslinking pathway, density functional theory (DFT) calculations were performed on a model system comprising a polyimine fragment containing two repeating units to represent the 1D precursor and a diamine crosslinker. For this model system, the structures of the reactants and products were optimized, and transition‐state searches were carried out for the key bond‐forming step, yielding the corresponding activation barriers and reaction free energies (Figure 4b). The initial nucleophilic attack of MPA on the protonated imine proceeds with a low activation free‐energy barrier of 3.25 kcal/mol. At the transition state, the distance between the nucleophilic nitrogen of MPA and the electrophilic imine carbon decreases from 3.02 Å in the reactant to 2.07 Å, reflecting the tendency to form a C─N bond. In the product (intermediate 1), this distance further shortens to 1.69 Å, accompanied by a reaction free energy of 1.82 kcal/mol, indicating the formation of a stable C─N bond and the aminal linkage. In contrast, using PPA as the crosslinker results in a substantially higher activation barrier of 19.15 kcal/mol and a less stable product (12.85 kcal/mol), consistent with experimental observations that PPA does not promote crosslinking. This can be attributed to the linear para‐geometry of PPA, which positions the amine groups too far apart to effectively approach and react with the protonated imine. As a result, the first nucleophilic attack is both geometrically and energetically unfavorable, explaining why no aminal formation occurs experimentally with PPA. The subsequent reaction of the remaining amine in intermediate 1 with a second protonated polyimine fragment to form the crosslinked intermediate 2 exhibits much higher activation barriers (66.96 and 69.43 kcal/mol for MPA and PPA, respectively), reflecting steric hindrance and conformational constraints associated with aligning two polymer chains for bond formation (Figure S16). The similar high barriers indicate that this second step is less sensitive to crosslinker structure. Collectively, these results demonstrate that the formation of aminal‐linked POPs is primarily governed by the initial nucleophilic addition step, which is highly sensitive to crosslinker geometry.

### 2.3 Applications in Hydrocarbon Separation

**FIGURE 5:**
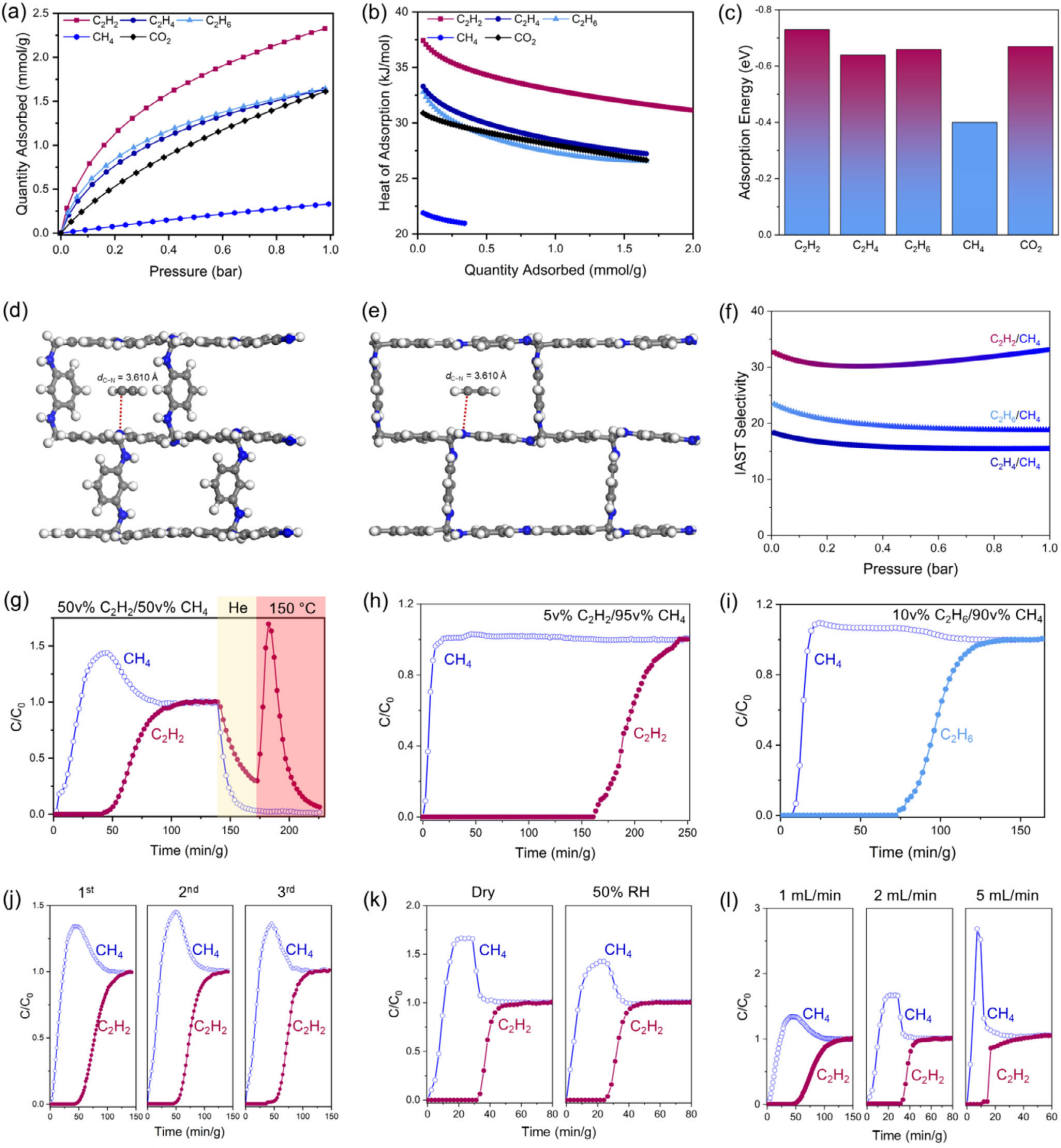
Gas adsorption and separation performance of TPA–PPA/MPA POP. (a) Adsorption isotherms of CH_4_, C_2_H_6_, C_2_H_4_, C_2_H_2_, and CO_2_ measured at 298 K in the pressure range of 0–1 bar. (b) Isosteric heats of adsorption for CH_4_, C_2_H_6_, C_2_H_4_, C_2_H_2_, and CO_2_. (c) Adsorption energies of CH_4_, C_2_H_6_, C_2_H_4_, C_2_H_2_, and CO_2_ on the POP obtained from DFT calculations. (d, e) Optimized adsorption configurations of C_2_H_2_ on the POP, showing the distances between the POP framework and the gas molecules from different views. (f) IAST selectivities for C_2_H_2_/CH_4_, C_2_H_4_/CH_4_, and C_2_H_6_/CH_4_ gas mixtures (50/50, v/v), calculated using ideal adsorbed solution theory (IAST). Dynamic breakthrough curves for separation of gas mixtures: (g) C_2_H_2_/CH_4_ (50/50, v/v), (h) C_2_H_2_/CH_4_ (5/95, v/v), and (i) C_2_H_6_/CH_4_ (10/90, v/v) at 298 K with a gas mixture flow rate of 1 mL/min. (j) Three consecutive dynamic breakthrough cycles for separation of C_2_H_2_/CH_4_ (50/50, v/v) at a flow rate of 1 mL/min. (k) Dynamic breakthrough curves for separation of C_2_H_2_/CH_4_ (50/50, v/v) under dry and 50% relative humidity conditions at a flow rate of 2 mL/min. (l) Dynamic breakthrough curves for separation of C_2_H_2_/CH_4_ (50/50, v/v) at different flow rates of 1, 2, and 5 mL/min. All breakthrough experiments were performed at 298 K.

Given their relatively high surface area and rich microporosity, the synthesized POPs were envisioned as promising sorbents for hydrocarbon separation and purification. Among all samples, the TPA–PPA/MPA POP, which exhibited the highest BET specific surface area and a suitable nanoflake structure to facilitate mass transport, was selected for detailed adsorption studies. The adsorption capacities toward different gases—including methane (CH_4_), ethane (C_2_H_6_), ethylene (C_2_H_4_), acetylene (C_2_H_2_), and carbon dioxide (CO_2_)—were evaluated by measuring adsorption isotherms in the pressure range of 0–1 bar at 273, 283, and 298 K (Figure S17). The POP exhibited curved isotherms for C_2_H_2_, C_2_H_4_, C_2_H_6_, and CO_2_, with relatively high adsorption capacities of 2.37, 1.66, 1.67, and 1.66 mmol/g, respectively, at 1 bar and 298 K (Figure 5a), indicating strong interactions with these molecules due to their higher polarizability and quadrupole interactions with the framework. Specifically, the highest C_2_H_2_ uptake can be attributed to its small kinetic diameter, linear geometry, and relatively high polarizability, which allow it to efficiently access and occupy the micropores of the POP framework. In contrast, the adsorption capacity for CH_4_ was significantly lower (0.37 mmol/g) under the same conditions, reflecting weak interactions with the POP framework, which can be explained by the low polarizability and absence of a quadrupole moment of CH_4_. Notably, the 1D TPA–PPA polymer precursor with a low surface area showed negligible gas uptake (0.01–0.14 mmol/g) under the same conditions (Figure S18), highlighting the crucial role of microporosity in governing adsorption performance.

The heat of adsorption of each gas was calculated from adsorption isotherms measured at multiple temperatures using the Clausius–Clapeyron equation to evaluate the binding affinity of the gases toward the POP. C_2_H_2_ exhibited the highest isosteric heat of adsorption (*Q*_s__t_ = 37.4 kJ/mol), slightly higher than those of C_2_H_6_, C_2_H_4_, and CO_2_ (30.9–33.3 kJ/mol), and substantially higher than that of CH_4_ (21.9 kJ/mol) (Figure 5b). These observations are qualitatively supported by DFT calculations (Figures 5c–e and S19). C_2_H_2_ exhibits the strongest interaction with the POP, with an adsorption energy (***E*_a_**) of approximately −0.73 eV and a C(C_2_H_2_)···N(aminal) distance of ∼3.6 Å, suggesting π–lone pair and quadrupole–polar interactions between C_2_H_2_ and the aminal sites. In comparison, C_2_H_4_, C_2_H_6_, and CO_2_ show slightly weaker interactions, with adsorption energies ranging from −0.64 to −0.67 eV, while CH_4_ exhibits the weakest interaction (***E*_a_** = −0.40 eV). The DFT results show the same qualitative trend as the experimental adsorption heats and adsorption isotherms, indicating stronger interactions of the POP with C_2_ hydrocarbons and CO_2_ than with CH_4_, and highlighting its potential as a selective sorbent for hydrocarbon separation and upgrading.

The potential of the POP for hydrocarbon separation was firstly evaluated by calculating the selectivity for different gas mixtures using the single‐component adsorption isotherms and the Ideal Adsorbed Solution Theory (IAST) (Figures 5f and S20; and Table S3). Remarkably, the TPA–PPA/MPA POP exhibited high selectivities for C_2_H_2_/CH_4_, C_2_H_6_/CH_4_, and C_2_H_4_/CH_4_, reaching 32.6, 23.4, and 18.3, respectively, for equimolar (50:50 v/v) mixtures at 298 K. These results indicate that the POP preferentially adsorbs C_2_ hydrocarbons over CH_4_, highlighting its potential as an effective sorbent for hydrocarbon separation and purification. Notably, both the C_2_H_2_ uptake and the corresponding selectivities are comparable to those reported for state‐of‐the‐art porous organic materials, demonstrating the competitive performance of TPA–PPA/MPA POP (Table S4) [58, 59, 60, 61, 62, 63]. Furthermore, the material also showed relatively high C_2_H_2_/CO_2_ and CO_2_/CH_4_ selectivities of up to 4.9 and 7.9, respectively (Figure S21), suggesting additional potential for hydrocarbon upgrading through removal of CO_2_ impurities.

To validate the separation performance predicted from the single‐gas adsorption results, dynamic breakthrough experiments were conducted using the POP as a fixed‐bed sorbent. In a typical experiment, 500 mg of TPA–PPA/MPA POP was packed into a column, and gas mixtures containing C_2_H_2_ and CH_4_ with different compositions were passed through the column. When a 50/50 (v/v) C_2_H_2_/CH_4_ mixture was introduced at a flow rate of 1 mL/min, CH_4_ eluted rapidly within a few minutes due to its low adsorption capacity (Figure 5g). In contrast, no C_2_H_2_ breakthrough was observed until 43.2 min/g (corresponding to 21.6 min with 0.5 g sorbent), indicating that C_2_H_2_ was efficiently captured by the POP column and that the gas eluting from the column during the initial 43.2 min/g consisted of pure CH_4_, demonstrating effective purification of the gas mixture. After the column reached its saturated adsorption capacity, helium was flushed through the column, during which CH_4_ and a small portion of the adsorbed C_2_H_2_ were released. Subsequent thermal activation at 150°C resulted in the complete desorption of the remaining C_2_H_2_, enabling the recovery of high‐purity C_2_H_2_. The separation performance was even more pronounced for gas mixtures with lower C_2_H_2_ concentrations. For a 5/95 (v/v) C_2_H_2_/CH_4_ mixture, CH_4_ eluted rapidly, whereas C_2_H_2_ began to break through at 163.2 min/g (corresponding to 81.6 min for 0.5 g of sorbent), indicating that the effluent during this period was predominantly CH_4_ (Figure 5h). Similarly, the POP column demonstrated high separation efficiency for C_2_H_6_/CH_4_, C_2_H_4_/CH_4_, and CO_2_/CH_4_ mixtures (Figures 5i and S22). For a 10/90 (v/v) C_2_H_6_/CH_4_ mixture, CH_4_ eluted quickly, while C_2_H_6_ started to break through at 74.4 min/g (corresponding to ∼37.2 min for 0.5 g of sorbent).

Furthermore, the POP column exhibited excellent recyclability in breakthrough experiments. Consecutive measurements showed no significant loss in separation performance for C_2_H_2_/CH_4_ mixtures, as the C_2_H_2_ retention time remained essentially unchanged over at least 10 cycles (Figures 5j and S23). Between each cycle, the column was regenerated by heating at 150°C. The POP column also maintained high separation performance under humid conditions; in the presence of 50% relative humidity, the C_2_H_2_ retention time showed only a slight decrease compared with that observed for dry gas mixtures, which can be attributed to the relatively hydrophobic nature of the POP framework and the much faster adsorption kinetics of C_2_H_2_ compared with water (Figure 5k). Finally, the POP column demonstrated consistently high separation performance at elevated flow rates: increasing the flow rate from 1 to 2 and 5 mL/min did not significantly reduce the separation efficiency (Figure 5l). These results collectively demonstrate the efficient separation of C_2_ hydrocarbons from CH_4_ using the POP sorbent and highlight its strong potential for natural gas upgrading applications.

## 3 Conclusion

In conclusion, we present a new strategy for synthesizing POPs through the crosslinking of linear polyimines via nucleophilic addition reactions between amines and imines to form aminal linkages. The formation of aminal linkages was confirmed by infrared spectroscopy and solid‐state ^13^C NMR spectroscopy, and the proposed mechanism was further supported by density functional theory (DFT) calculations. This approach demonstrates good generality, as a range of linear polyimines can be effectively transformed into porous networks. Compared with the low surface area of the precursor linear polyimines, the resulting aminal‐linked POPs exhibit markedly enhanced surface areas and abundant microporosity. The obtained POPs show high uptake of C_2_ hydrocarbons and high selectivity over CH_4_, as evidenced by dynamic breakthrough experiments, demonstrating their promise for hydrocarbon separation. Beyond enriching the synthetic toolbox for POP materials, this method will also offer a versatile platform for post‐synthetic modification, structural modulation, and functionalization of diverse polyimine‐based polymers, including imine‐linked COFs, thereby enabling the development of advanced adsorbents, separation membranes, and catalytic supports with tailored properties. Furthermore, their cost‐effectiveness will greatly facilitate large‐scale production and industrial applications.

## Author Contributions

**Xuejie Li**: methodology, investigation, and validation. **Yayu Yan**: methodology, investigation, and validation. **Qiaohong Li**: conceptualization, methodology, investigation, validation, software, and supervision. **Michelle Åhlén**: methodology, investigation, writing – review and editing, visualization, and formal analysis. **Chao Xu**: conceptualization, methodology, data curation, investigation, validation, formal analysis, supervision, resources, project administration, funding acquisition, writing – original draft, writing – review and editing, visualization, and software.

## Conflicts of Interest

The authors declare no conflicts of interest.

## Supporting information




**Supporting File 1**: anie73362‐sup‐0001‐SuppMat.pdf.

## Data Availability

The data that support the findings of this study are available from the corresponding author upon reasonable request.
