# Supplementary material for: Crosslinking of Linear Polyimines Into Aminal‐Linked Porous Organic Polymers for C2 Hydrocarbon/Methane Separation
Source: Angew Chem Int Ed Engl. 2026 Jun 24;65(35):e5692086. doi: 10.1002/anie.5692086 (PMC13502617; doi:10.1002/anie.5692086)
Supplement: Supplementary file 1 [file ANIE-65-e5692086-s001.pdf]

Supporting information

Crosslinking of Linear Polyimines into Aminal-Linked Porous Organic Polymers for Efficient C_2 Hydrocarbon/Methane Separation

Xuejie Li[a], Yayu Yan[b], Qiaohong Li[b]*, Michelle Åhlén[c], Chao Xu[c]*

a. Institute of Molecular Engineering and Applied Chemistry, Anhui University of Technology, Ma'anshan, 243002, P. R. China

b. State Key Laboratory of Structural Chemistry, Fujian Institute of Research on the Structure of Matter, Chinese Academy of Sciences Fuzhou, Fujian 350002, P. R. China. E-mail: lqh2382@fjirsm.ac.cn

c. Division of Nanotechnology and Functional Materials, Department of Materials Science and Engineering, Ångström Laboratory, Uppsala University, SE-751 21 Uppsala, Sweden. E-mail: chao.xu@angstrom.uu.se

1. Materials

Terephthalaldehyde (TPA), *p*-phenylenediamine (PPA), *m*-phenylenediamine (MPA), 4,4′-biphenyldicarboxaldehyde (BDA), and benzidine (BZD) were purchased from Alfa Aesar. All solvents were obtained from Sinopharm Chemical Reagent Co. Ltd. All chemicals and solvents were used as received without further purification.

Synthesis

Synthesis of TPA-PPA polymer

Terephthalaldehyde (TPA; 1 mmol, 134 mg) was added to 5 mL of ethanol (EtOH), followed by the addition of 2 mL of an aqueous acetic acid solution (AcOH, 3 M). The mixture was stirred for 20 min until a clear solution was obtained. Meanwhile, *p*-phenylenediamine (PPA; 1 mmol, 108 mg) was dissolved in 5 mL of EtOH. The PPA solution was then added dropwise to the TPA solution under stirring, resulting in the immediate formation of a yellow precipitate. The reaction mixture was stirred at room temperature for 3 days. The resulting yellow precipitate was collected by filtration and thoroughly washed with water, ethanol, and acetone. The product was further purified by Soxhlet extraction using ethanol for 12 h, followed by drying in an oven at 100 °C for 12 h. The final yellow product was denoted as TPA–PPA polymer. Yield: 98 % (202 mg).

Synthesis of TPA-BZD polymer

The synthesis was carried out following the same procedure as for the TPA–PPA polymer, with benzidine (BZD; 1 mmol, 184 mg) used instead of PPA. Yield: 92 % (259 mg).

Synthesis of BDA-PPA polymer

The synthesis was carried out following the same procedure as for the TPA–PPA polymer, with 4,4′-biphenyldicarboxaldehyde (BDA; 1 mmol, 210 mg) used instead of TPA. Yield: 90 % (254 mg).

Synthesis of BDA-BZD polymer

The synthesis was carried out following the same procedure as for the TPA–PPA polymer, with 4,4′-biphenyldicarboxaldehyde (BDA; 1 mmol, 210 mg) and benzidine (BZD; 1 mmol, 184 mg) used instead of TPA and PPA, respectively. Yield: 88 % (315 mg).

Synthesis of TPA-PPA/MPA POP

The TPA–PPA/MPA POP was synthesized *via* a crosslinking reaction between *m*-phenylenediamine (MPA) and TPA–PPA polymer under optimized conditions to achieve high surface area and yield. Specifically, the effects of solvent, type and concentration of acid catalyst, reaction temperature, and the molar ratio between TPA–PPA polymer and MPA on the surface area were systematically investigated. Details of the synthesis optimization and the results are summarized in Table S1.

Taking the optimized conditions as an example (entry 2 in Table S1), TPA–PPA polymer (0.5 mmol, 103 mg) was added to 2.5 mL of dioxane. An aqueous solution of trifluoroacetic acid (TFA, 0.5 mL, 6 M) was added, and the mixture was stirred at room temperature for 20 min to form a homogeneous suspension. Meanwhile, MPA (0.5 mmol, 54 mg) was dissolved in 2.5 mL of dioxane to form a clear brown solution. The MPA solution was then added dropwise to the TPA–PPA suspension. The resulting mixture was stirred at room temperature for 20 min, transferred to a sealed autoclave, and heated in an oven at 120 °C for 3 days. The resulting brown precipitate was collected by filtration, thoroughly washed with water, ethanol, and acetone, and further purified by Soxhlet extraction using methanol for 12 h. The product was dried in an oven at 100 °C for 12 h to yield a brown powder, denoted as TPA–PPA/MPA POP. Yield: 85.4% (134 mg).

Synthesis of TPA-BZD/MPA POP

The synthesis was carried out following the same procedure as for TPA–PPA/MPA POP under the optimized conditions, with TPA–BZD polymer (0.5 mmol, 141 mg) used instead of TPA–PPA polymer, yielding a brown powder. Yield: 81.5% (159 mg).

Synthesis of BDA-PPA/MPA POP

The synthesis was carried out following the same procedure as for TPA–PPA/MPA POP under the optimized conditions, with BDA-PPA polymer (0.5 mmol, 141 mg) used instead of TPA–PPA polymer, yielding a brown powder. Yield: 86.1% (168 mg).

Synthesis of BDA-BZD/MPA POP

The synthesis was carried out following the same procedure as for TPA–PPA/MPA POP under the optimized conditions, with BDA-BZD polymer (0.5 mmol, 179 mg) used instead of TPA–PPA polymer, yielding a brown powder. Yield: 83.2% (194 mg).

Synthesis of TPA-PPA/PPA POP

The synthesis was carried out following the same procedure as for TPA–PPA/MPA POP under the optimized conditions, with PPA (0.5 mmol, 54 mg) used instead of MPA. The resulting product retained the yellow color of the TPA–PPA polymer, suggesting that no significant crosslinking reaction occurred. Yield: 59% (93 mg).

Large-Scale Synthesis of TPA–PPA/MPA POP

The large-scale synthesis of TPA–PPA/MPA POP was adapted from the standard synthetic procedure described above, with modifications to the reaction scale and conditions. The amounts of reactants and catalyst were increased by a factor of 100, and the solvent volume was increased by a factor of 50. Unlike the standard synthesis, which was conducted in a sealed autoclave and heated in an oven without stirring, the large-scale synthesis was carried out in a round-bottom flask under continuous stirring and reflux to ensure a more homogeneous reaction mixture and improved mass transfer.

Specifically, TPA–PPA polymer (50 mmol, 10.3 g) was dispersed in 125 mL of dioxane and charged into a 500 mL round-bottom flask. An aqueous solution of trifluoroacetic acid (TFA, 50 mL, 6 M) was then added, and the mixture was stirred at room temperature for 20 min to form a homogeneous suspension. Meanwhile, MPA (50 mmol, 5.4 g) was dissolved in 125 mL of dioxane to obtain a clear brown solution. The MPA solution was subsequently added to the TPA suspension. The resulting reaction mixture was heated under reflux at 120 °C with continuous stirring and a water-cooled condenser for 3 days.

The resulting brown precipitate was collected by filtration, thoroughly washed with water, ethanol, and acetone, and further purified by Soxhlet extraction with acetone for 12 h. The product was then dried in an oven at 100 °C for 12 h, affording a brown powder, denoted as Large-scale TPA–PPA/MPA POP. Yield: 98.6% (15.48 g).

Note: All 1D polymer precursors and the resulting crosslinked POPs are insoluble in common organic solvents.

2. Characterizations

Infrared (IR) spectra were recorded on a Bruker Tensor 27 spectrometer equipped with an attenuated total reflectance (ATR) accessory. Solid-state ^{13}C cross-polarization magic-angle spinning (CPMAS) nuclear magnetic resonance (NMR) spectra were collected on a Bruker AVWBIII600 spectrometer using a 4 mm MAS probe at a spinning rate of 14 kHz. Powder X-ray diffraction (PXRD) patterns were

obtained on a Rigaku Ultima IV diffractometer. X-ray photoelectron spectra (XPS) were recorded on a Thermo Scientific K-Alpha spectrometer. Scanning electron microscopy (SEM) images were acquired using a Carl Zeiss Merlin field-emission SEM (FE-SEM). Thermogravimetric analysis (TGA) was performed on a Mettler Toledo TGA/SDTA851e analyzer under a N_2 or air atmosphere (flow rate: 60 mL/min) from 25 to 800 °C with a heating rate of 10 °C/min.

All gas sorption isotherms were recorded using a Kubo-X1000 surface area and porosity analyzer. Prior to analysis, all samples were degassed at 120 °C under vacuum for 12 h. N_2 adsorption–desorption isotherms were measured at 77 K using a liquid nitrogen bath. Specific surface areas were calculated from adsorption data in the relative pressure range P/P_0 = 0.01–0.15 using the Brunauer–Emmett–Teller (BET) theory. Pore size distributions were determined from the N_2 adsorption isotherms using the density functional theory (DFT) model.

Carbon dioxide (CO_2) and hydrocarbon (acetylene (C_2H_2), ethylene (C_2H_4), ethane (C_2H_6), methane (CH_4)) adsorption isotherms were measured at 273, 288, and 298 K using a water bath. The heat of adsorption of the gases was calculated from isotherms measured at different temperatures using the Clausius–Clapeyron equation. Adsorption selectivity for gas mixtures was estimated based on single-gas adsorption isotherms using the ideal adsorbed solution theory (IAST). For IAST calculations, the adsorption isotherms were fitted using either the dual-site Langmuir or single-site Langmuir model. The fitted results are summarized in Table S3.

Dual-site Langmuir equation:

$$q = \frac{\left(q_{\{sat,A\}} * b_A * p\right)}{(1 + b_A * p)} + \frac{\left(q_{\{sat,B\}} * b_B * p\right)}{(1 + b_B * p)}$$

Single-site Langmuir equation:

$$q = \frac{\left(q_{\{sat\}} * b * p\right)}{(1 + b * p)}$$

where q is the adsorption capacity (mmol/g), q_{sat} is the saturation adsorption capacity (mmol/g), p is pressure (Pa), and b is Langmuir constant (Pa^{-1}), A and B are distinct adsorption sites.

Breakthrough measurements were performed using a CT-41 breakthrough analyzer (Xuzhou Beifang Gaorui Electronic Equipment Co., Ltd.) coupled to a GC-9860 gas chromatograph (Nanjing Hope Analytical Equipment Co., Ltd.) equipped with a thermal conductivity detector (TCD). Experiments were carried out at a pressure of 1 bar and a temperature of 25 °C. Approximately 500 mg of sample was tightly packed into a stainless-steel column and pretreated by heating in the chamber at 100 °C for 2 h prior to the measurement. Gas mixtures with desired compositions were prepared by blending individual pure gases using mass flow controllers (MFCs) and passed through the column at a controlled flow rate. The effluent gases were analyzed at the column outlet using the GC, and breakthrough curves were plotted based on the GC analysis results.

Theoretical calculations

Transition state search calculations and geometry optimizations were performed using the $DMol^3$ module in the Materials Studio software package. [1,2] The generalized gradient approximation (GGA) with the Perdew–Burke–Ernzerhof (PBE) functional [3] was employed to describe the exchange-correlation component of the Hamiltonian. Calculations used the double-numeric with polarization (DNP) basis set, [1,4,5] which is comparable in accuracy to 6-31G(d,p)**[6,7]. Numerical basis sets were applied to minimize basis-set superposition error. Orbital occupations were smeared with a value of 0.01 Hartree. Geometry optimizations and transition state searches were converged to tolerances of 2 ×

10^{-5} Hartree for energy, 0.01 Ha/Å for maximum force, and 0.01 Å for maximum displacement. Free energies and activation barriers were calculated from the optimized structures.

The adsorption energies of gases on the POP framework were calculated using density functional theory (DFT) as implemented in the Vienna Ab initio Simulation Package (VASP).[8] Electron–ion interactions were described using the Projector Augmented-Wave (PAW) method.[9] The generalized gradient approximation using the Perdew–Burke–Ernzerhof (GGA–PBE) functional was employed for the calculations.[10] The convergence criterion and plane-wave cutoff energy were set to 1×10^{-5} eV and 500 eV, respectively. Van der Waals (vdW) interactions were included using the Grimme DFT-D3 correction. Structural visualization and data processing were performed using VESTA[11] and VASPkit[12].

The adsorption energy (E_a) was calculated as:

$$E_a = E_{total} - E_{framework} - E_{gas}$$

It should be noted that the experimentally derived Q_{st} values and the DFT-calculated adsorption energies are not directly equivalent quantities and should therefore be compared qualitatively rather than quantitatively. The Q_{st} values represent differential adsorption enthalpies averaged over different adsorption sites and gas loadings in the real POP material, whereas the calculated adsorption energies correspond to the 0 K electronic adsorption energies of optimized gas molecules at representative binding sites in the model framework.

Table S1. Summary of synthesis conditions, yield, and surface area of TPA–PPA/MPA POP

Entry[a]	Polyimine /MPA ratio[b]	Acid catalyst[c]	Solvent[d]	Temp. (˚C)	Yield (%)	S_{BET} (m^2/g)
1	1:1	TFA (3 M)	Dioxane	120	80.2	472
2	1:1	TFA (6 M)	Dioxane	120	85.4	548
3	1:1	TFA (12 M)	Dioxane	120	83.4	477
4	1:1	AcOH (3 M)	Dioxane	120	77.7	314
5	1:1	AcOH (6 M)	Dioxane	120	78.9	346
6	1:1	AcOH (12M)	Dioxane	120	77.7	454
7	1:1	TFA (6 M)	THF	120	69.4	169
8	1:1	TFA (6 M)	Methanol	120	72.6	243
9	1:1	TFA (6 M)	Ethanol	120	75.2	273
10	1:0.8	TFA (6 M)	Dioxane	120	78.6	649
11	1:0.6	TFA (6 M)	Dioxane	120	76.8	511
12	1:0.4	TFA (6 M)	Dioxane	120	71.4	468
13	1:0.2	TFA (6 M)	Dioxane	120	65.9	324
14	1:1	TFA (6 M)	Dioxane	80	79.6	405
15	1:1	TFA (6 M)	Dioxane	60	77.1	160
16	1:1	TFA (6 M)	Dioxane	25	73.2	52

[a.] All reactions were carried out for 3 days.
[b.] Molar ratio between TPA–PPA polymer and MPA: 0.5 mmol of TPA–PPA polymer (103 mg) was used for all syntheses.
[c.] An aqueous solution of either trifluoroacetic acid (TFA) or acetic acid (AcOH) was used as the catalyst. The volume of TFA solution was 0.5 mL, while the volume of AcOH solution was 2 mL.
[d.] The total volume of solvent was 5 mL.

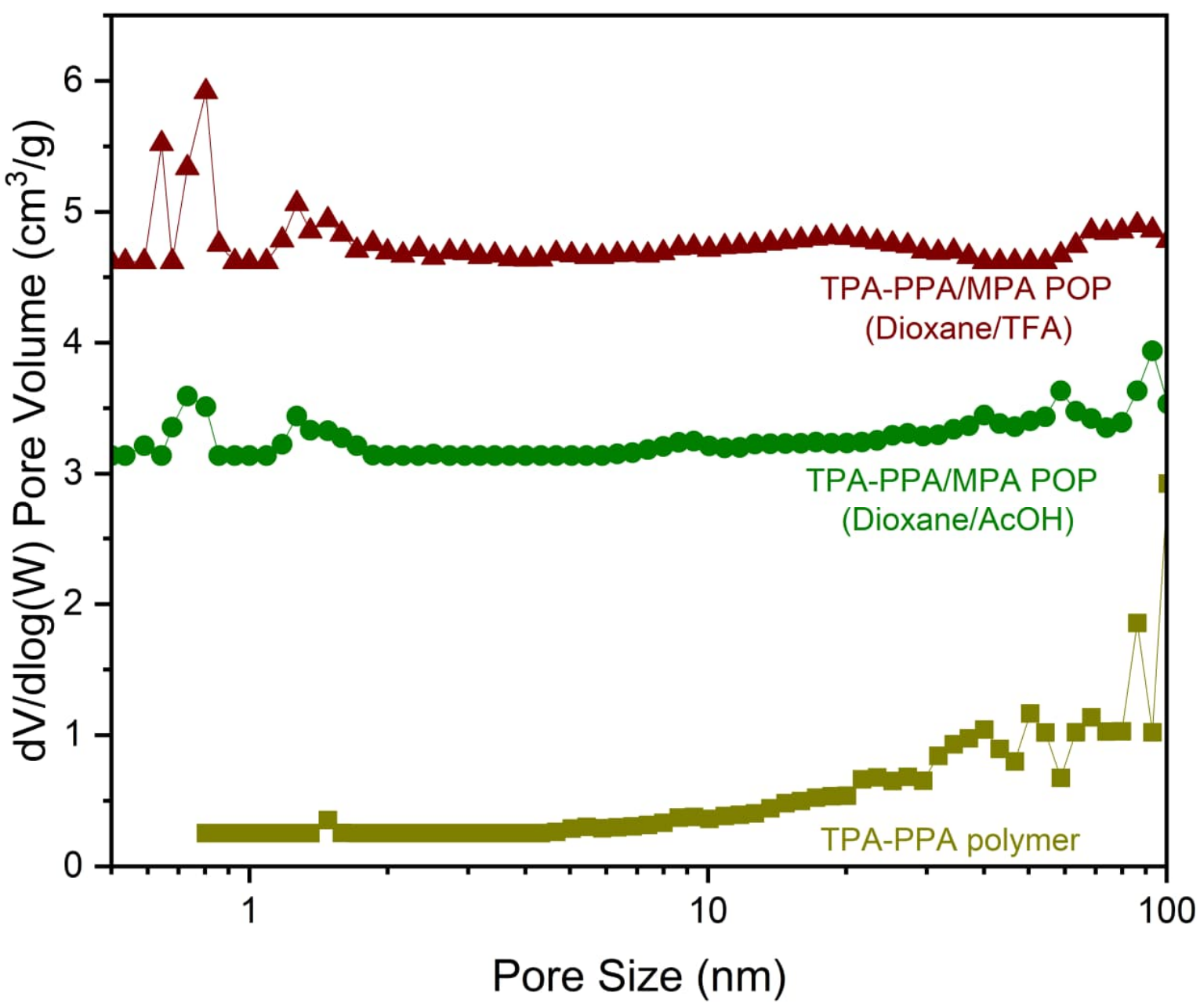

Figure S1. Pore size distribution of 1D TPA-PPA polymer and TPA-PPA/MPA POP.

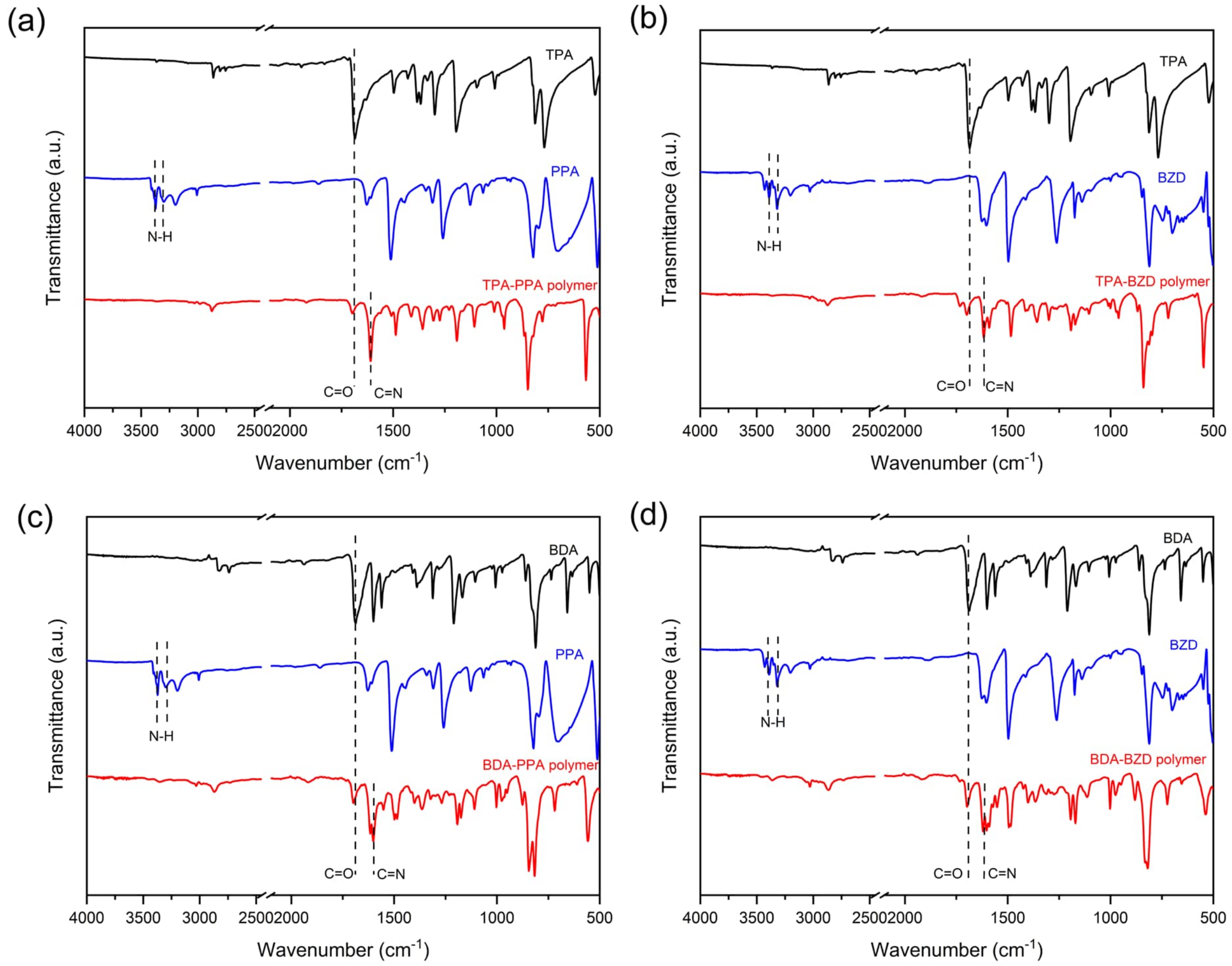

Figure S2. IR spectra of the 1D polyimines TPA–PPA, TPA–BZD, BDA–PPA, and BDA–BZD, together with the spectra of the corresponding monomers used for polymer synthesis.

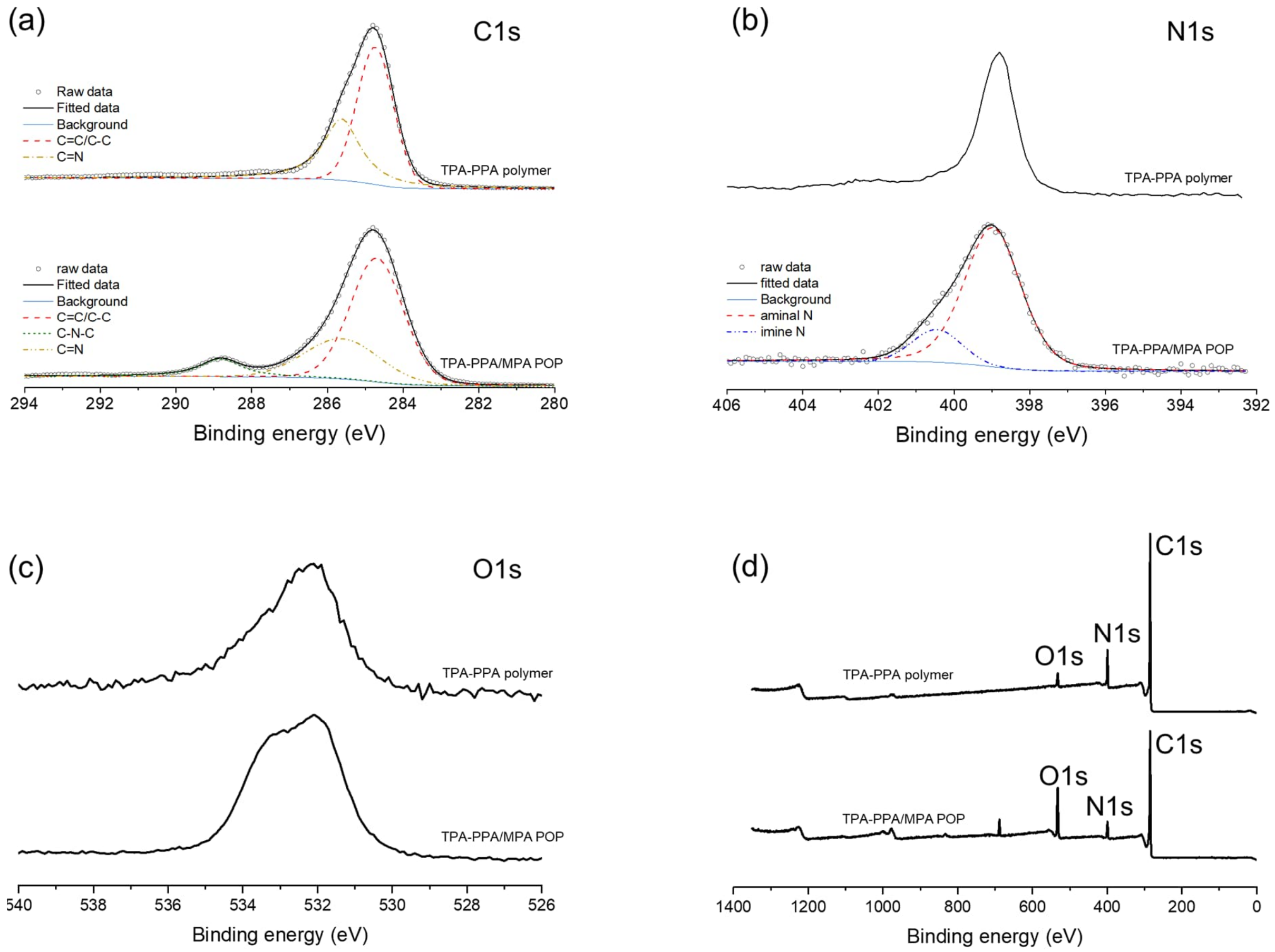

Figure S3. XPS of TPA–PPA polymer and TPA–PPA/MPA POP: (a) high-resolution C 1s, (b) N 1s, (c) O 1s, and (d) survey spectrum.

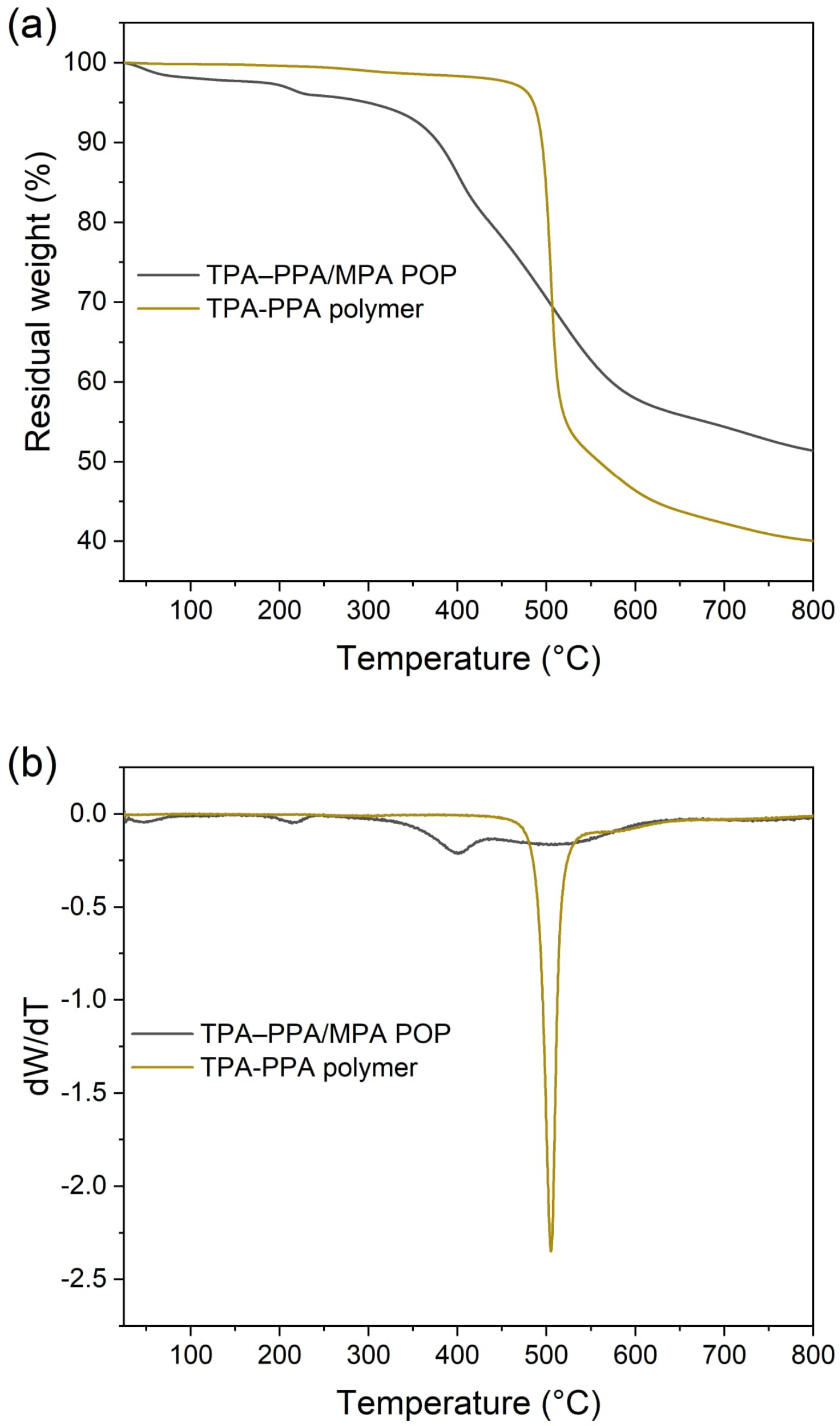

Figure S4. (a) TGA decomposition profiles of the 1D TPA–PPA polymer and TPA–PPA/MPA POP measured under an N_2 atmosphere; (b) first-derivative curves of the corresponding TGA profiles.

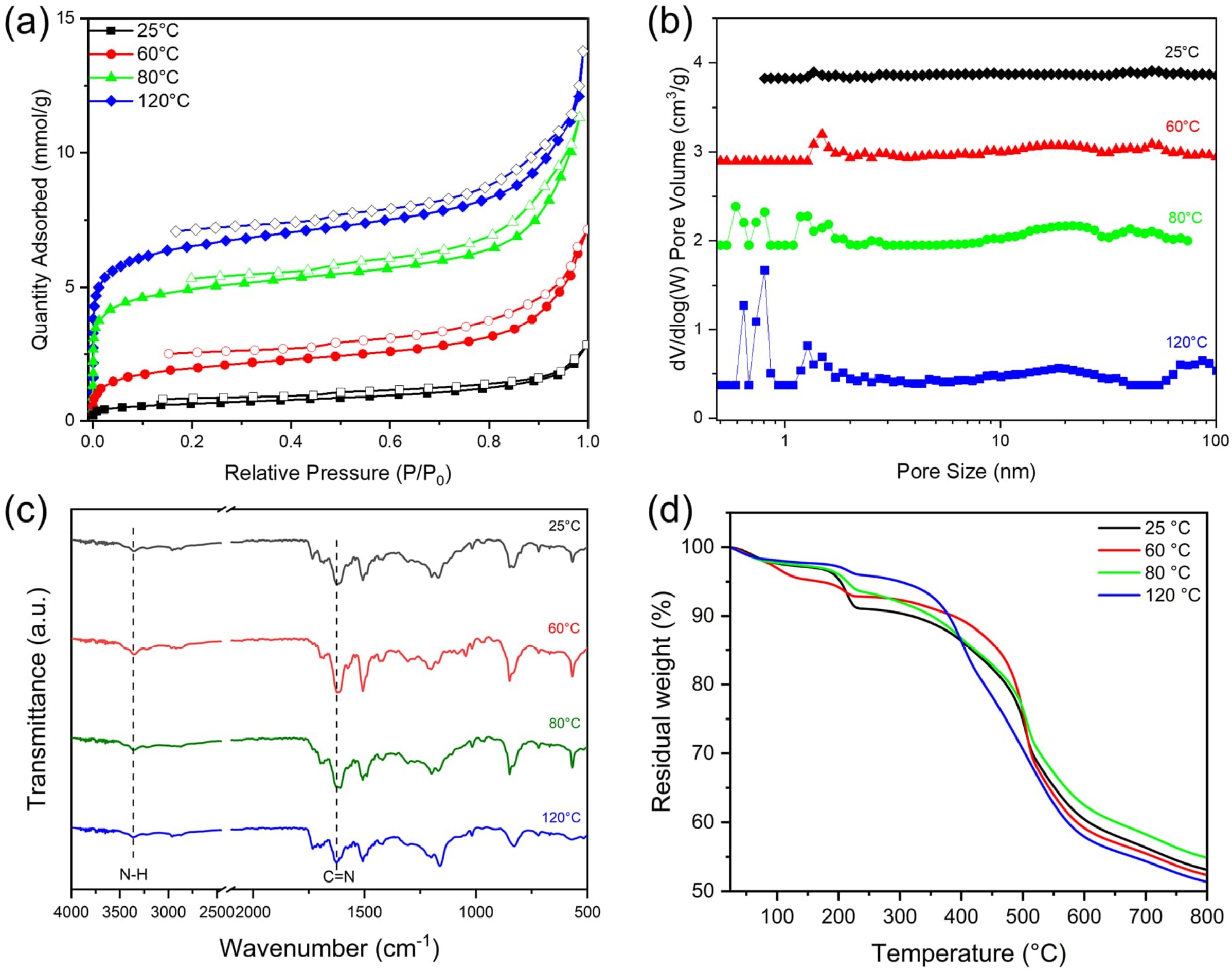

Figure S5. (a) N_2 sorption isotherms recorded at -196 °C (77 K), (b) pore size distributions, (c) IR spectra, (d) TGA decomposition profiles under an N_2 atmosphere of TPA-PPA/MPA POPs synthesized at different temperatures (25, 60, 80, and 120 °C). All reactions were carried out in dioxane using 6 M TFA as the catalyst for 3 days.

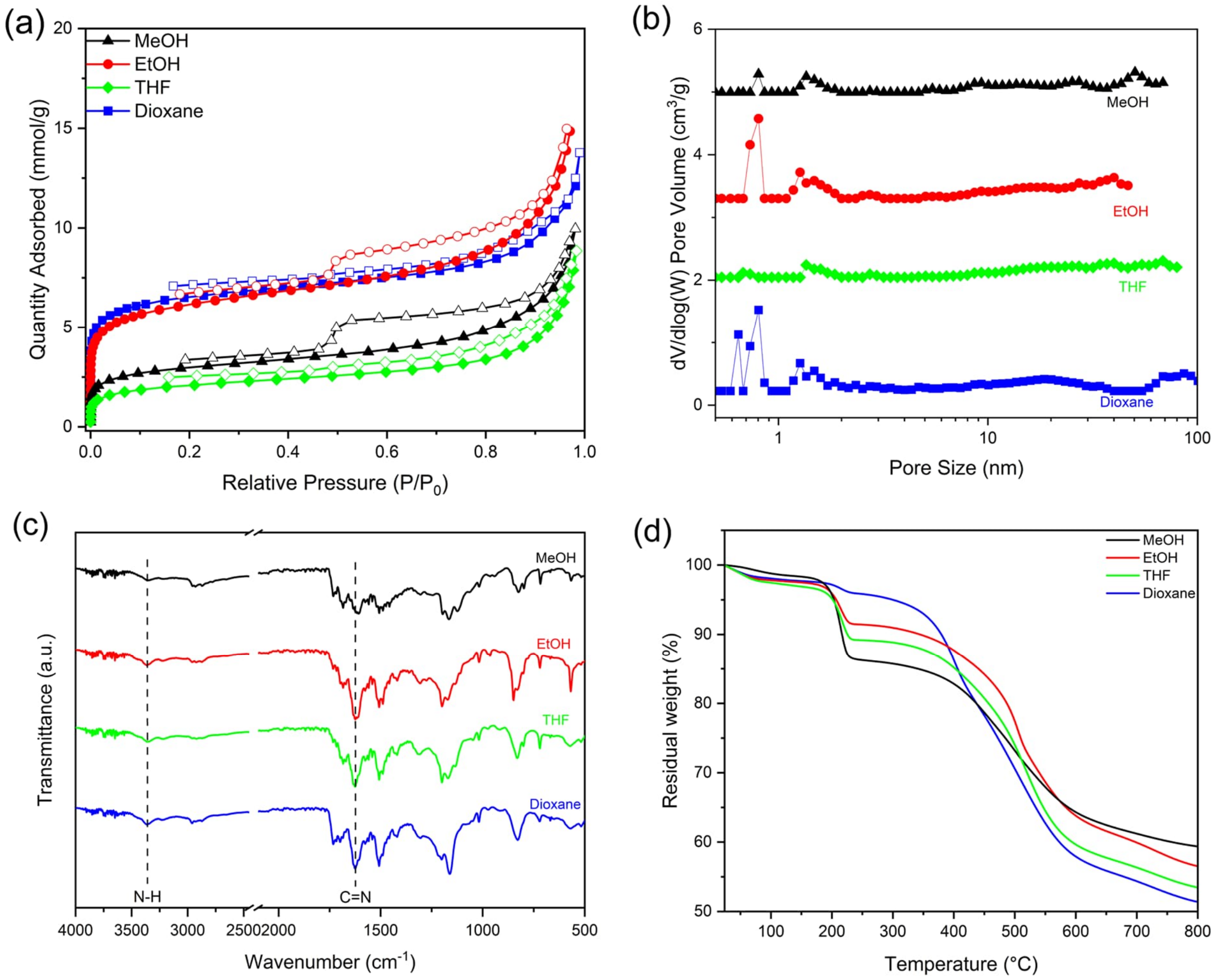

Figure S6. (a) N_2 sorption isotherms recorded at -196 °C (77 K), (b) pore size distributions, (c) IR spectra, and (d) TGA decomposition profiles under an N_2 atmosphere of TPA–PPA/MPA POPs synthesized in different solvents. All reactions were carried out using aqueous 6 M TFA as the catalyst at 120 °C for 3 days.

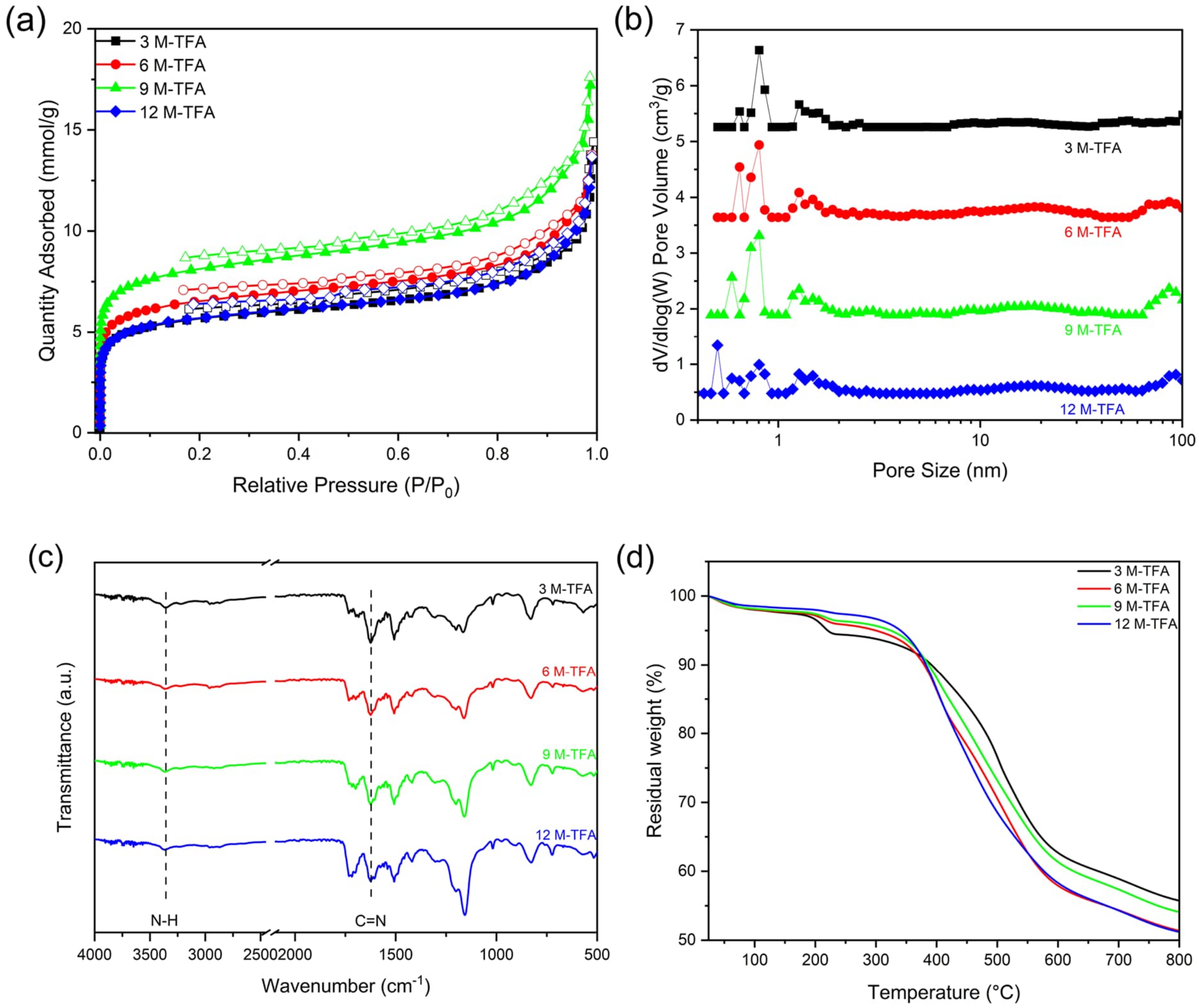

Figure S7. (a) N_2 sorption isotherms recorded at -196 °C (77 K), (b) pore size distributions, (c) IR spectra, and (d) TGA decomposition profiles under an N_2 atmosphere of TPA–PPA/MPA POPs synthesized in dioxane with TFA catalysts at varying concentrations. All reactions were conducted in dioxane at 120 °C for 3 days.

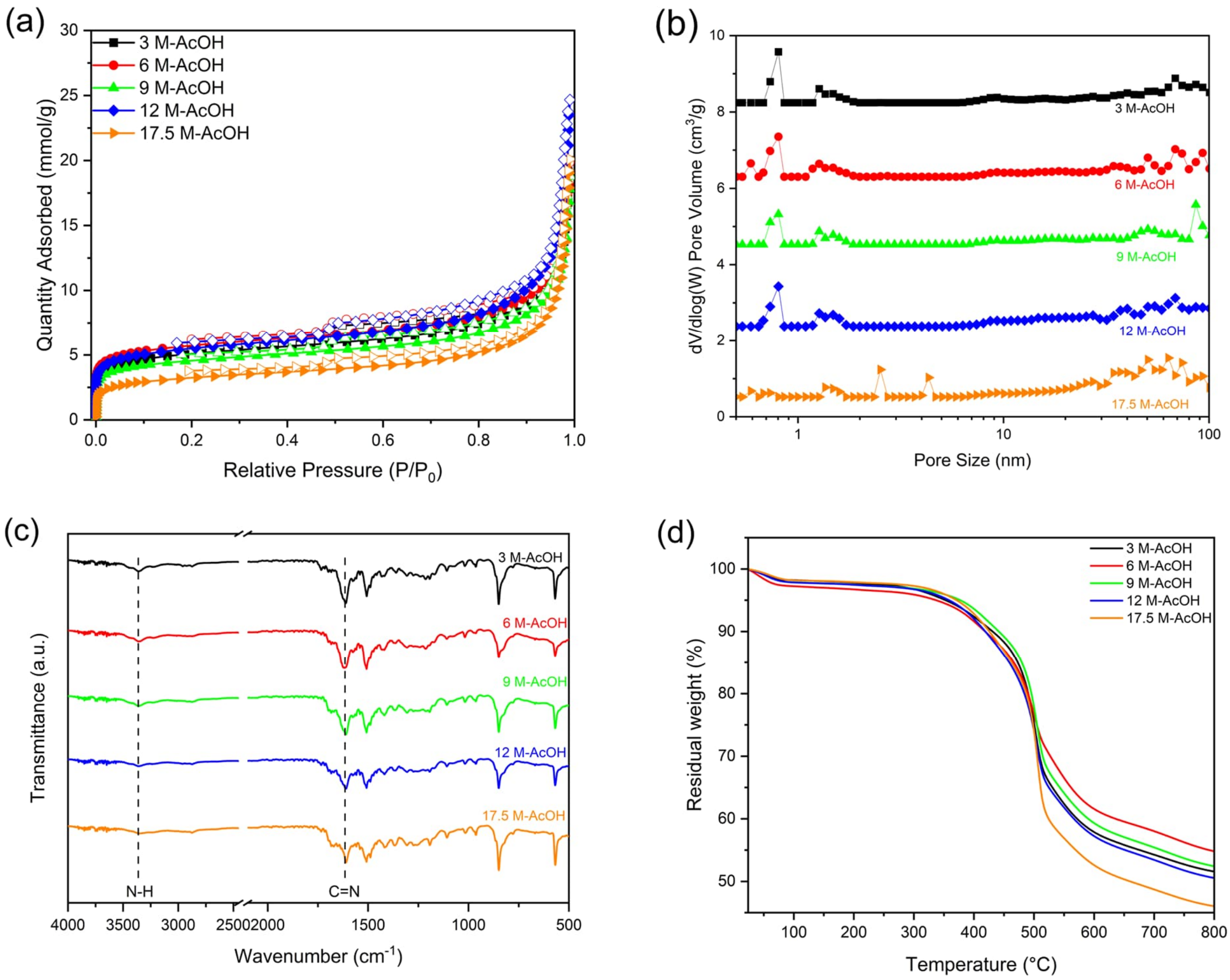

Figure S8. (a) N_2 sorption isotherms recorded at -196 °C (77 K), (b) pore size distributions, (c) infrared spectra, and (d) TGA decomposition profiles under an N_2 atmosphere of TPA–PPA/MPA POPs synthesized in dioxane with AcOH catalysts at varying concentrations. All reactions were conducted in dioxane at 120 °C for 3 days.

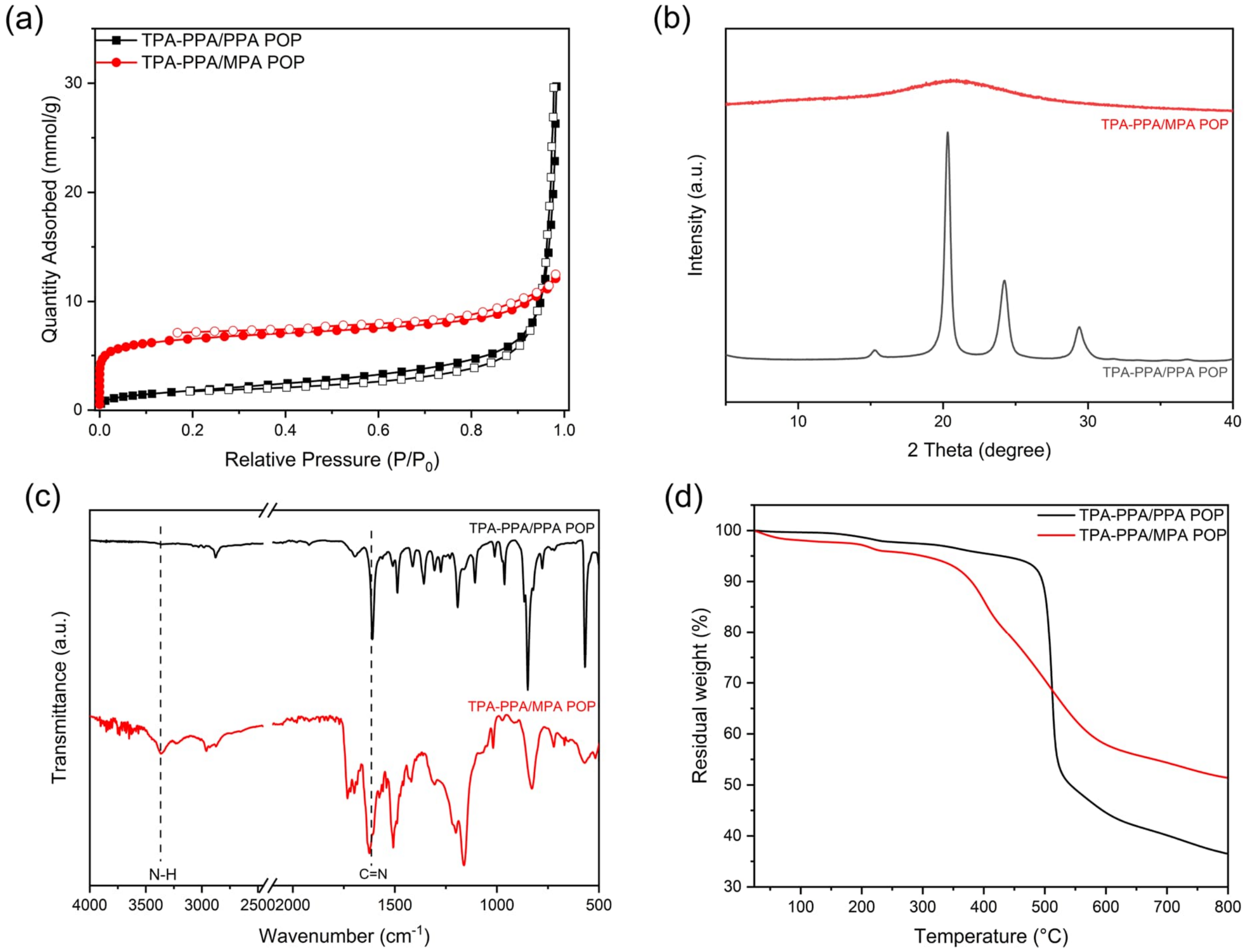

Figure S9. (a) N_2 sorption isotherms recorded at -196 °C (77 K), (b) PXRD patterns, (c) IR spectra, and (d) TGA decomposition profiles under an N_2 atmosphere of TPA–PPA/MPA and TPA–PPA/PPA POPs. All syntheses were carried out in dioxane using 6 M TFA as the catalyst at 120 °C for 3 days.

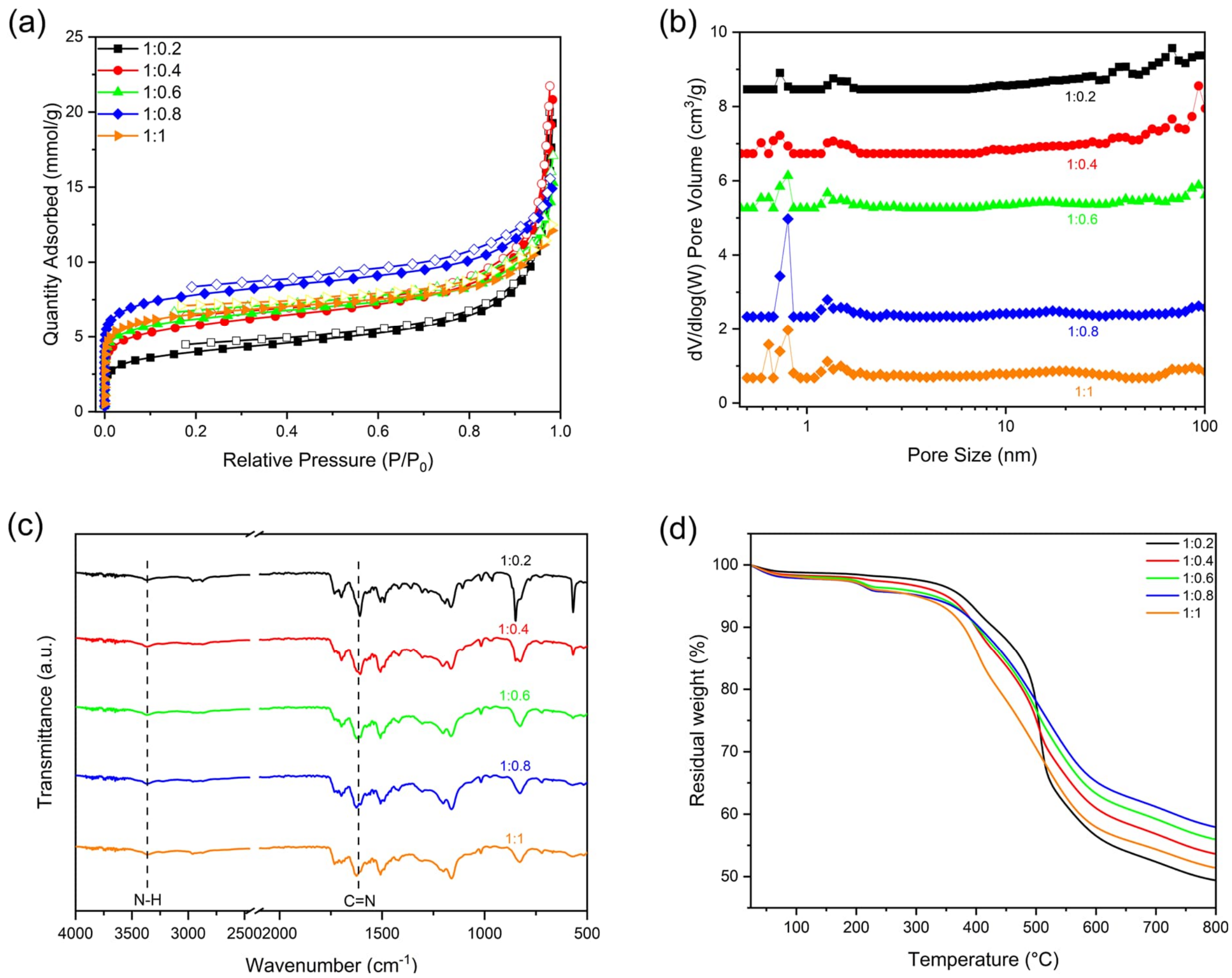

Figure S10. (a) N_2 sorption isotherms recorded at -196 °C (77 K), (b) pore size distributions, (c) IR spectra, and (d) TGA decomposition profiles under an N_2 atmosphere of TPA–PPA/MPA POPs synthesized with varying molar ratios of the TPA–PPA polymer and MPA crosslinker. All reactions were carried out in dioxane with 6 M TFA as the catalyst at 120 °C for 3 days.

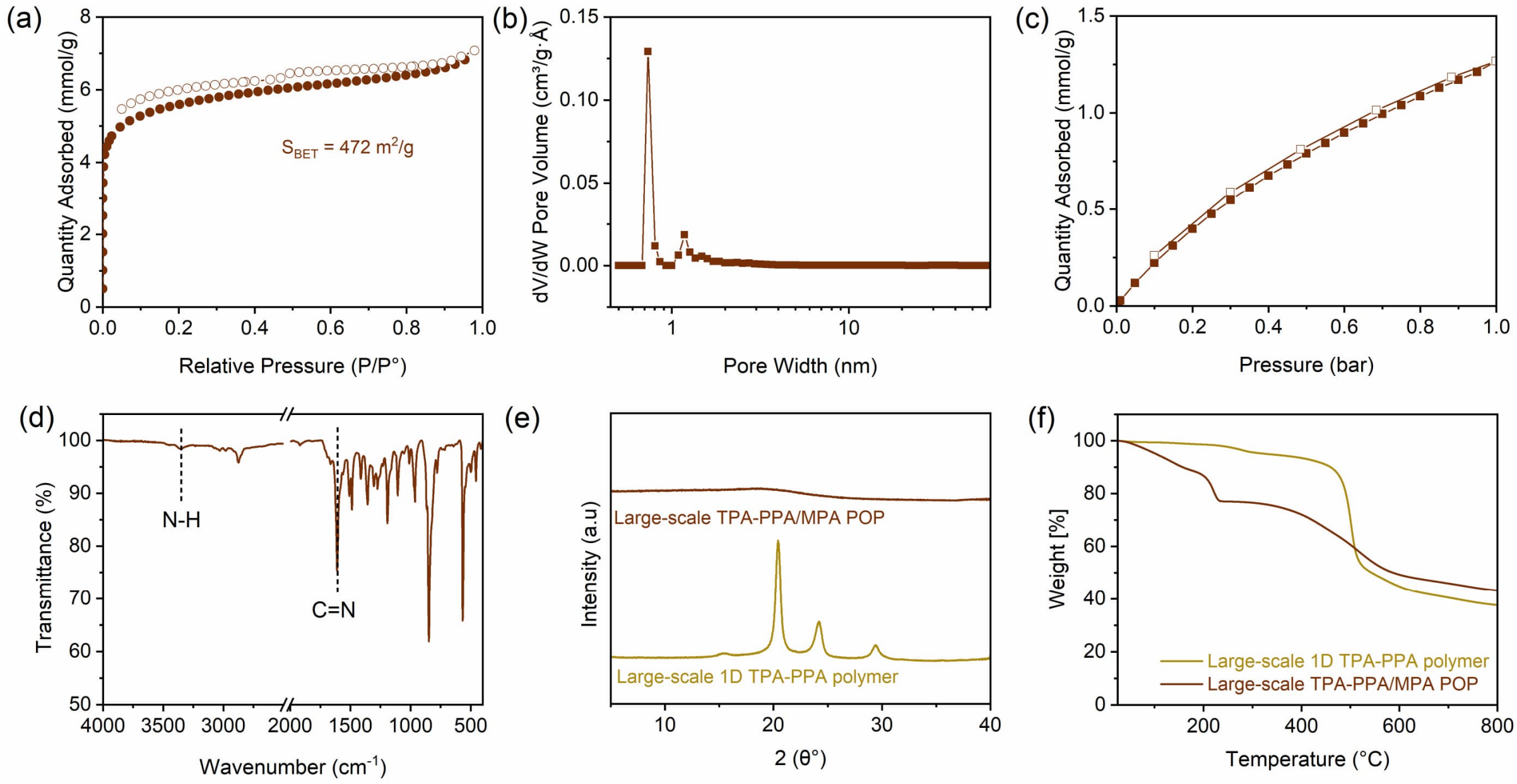

Figure S11. (a) N_2 sorption isotherm recorded at -196 °C (77 K), (b) pore size distribution, (c) CO_2 sorption isotherm recorded at 25 °C (298 K), and (d) FTIR spectra of large-scale synthesized TPA-PPA/MPA POP. (e) PXRD patterns and (f) TGA decomposition profiles recorded in N_2 atmosphere of large-scale synthesized 1D TPA-PPA polymer and TPA-PPA/MPA POP.

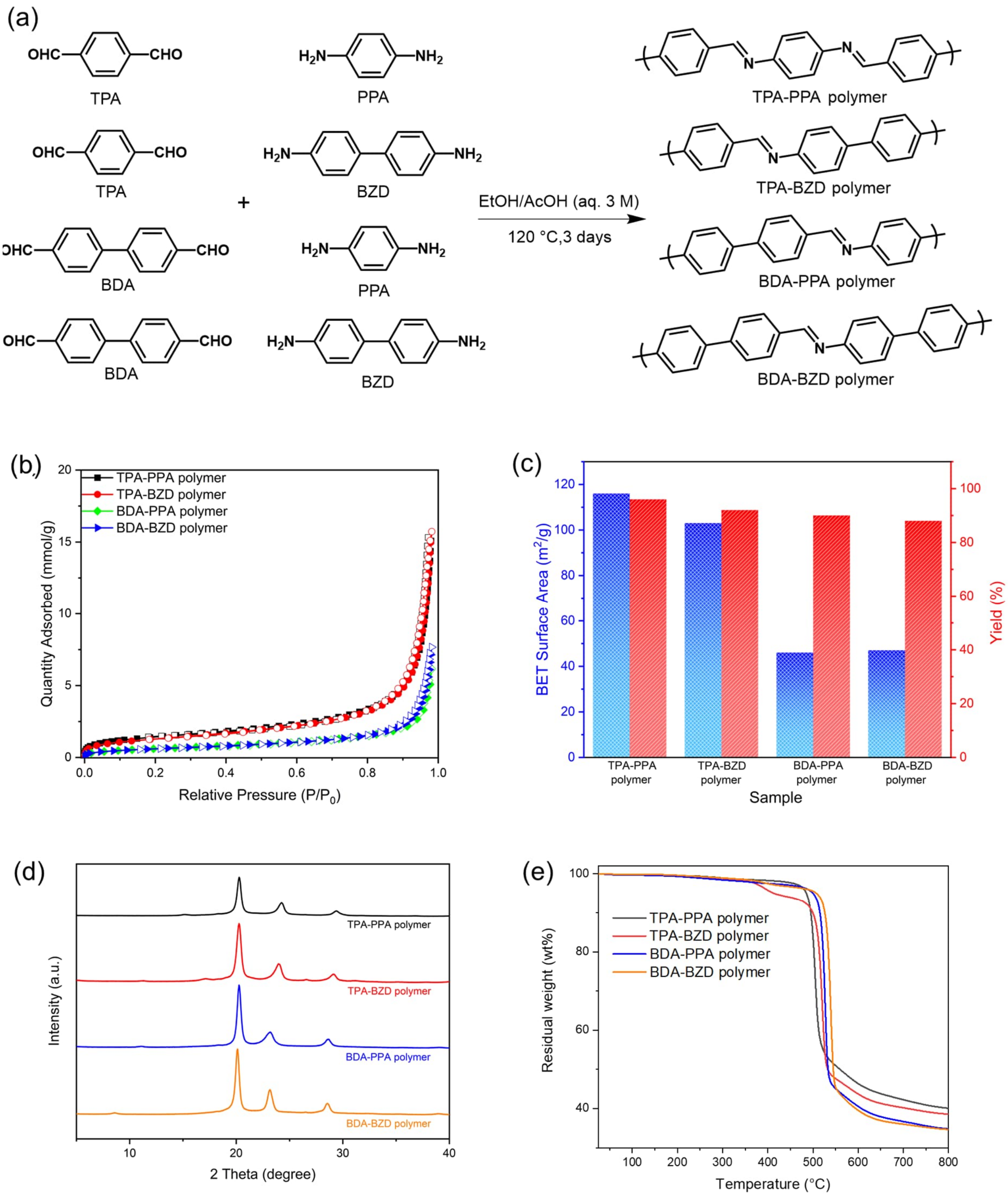

Figure S12. (a) Synthesis routes of different one-dimensional polyimine precursors. (b) N_2 sorption isotherms recorded at -196 °C (77 K), (c) comparison of BET surface areas and yields, (d) PXRD patterns, and (e) TGA decomposition profiles of the polyimine precursors.

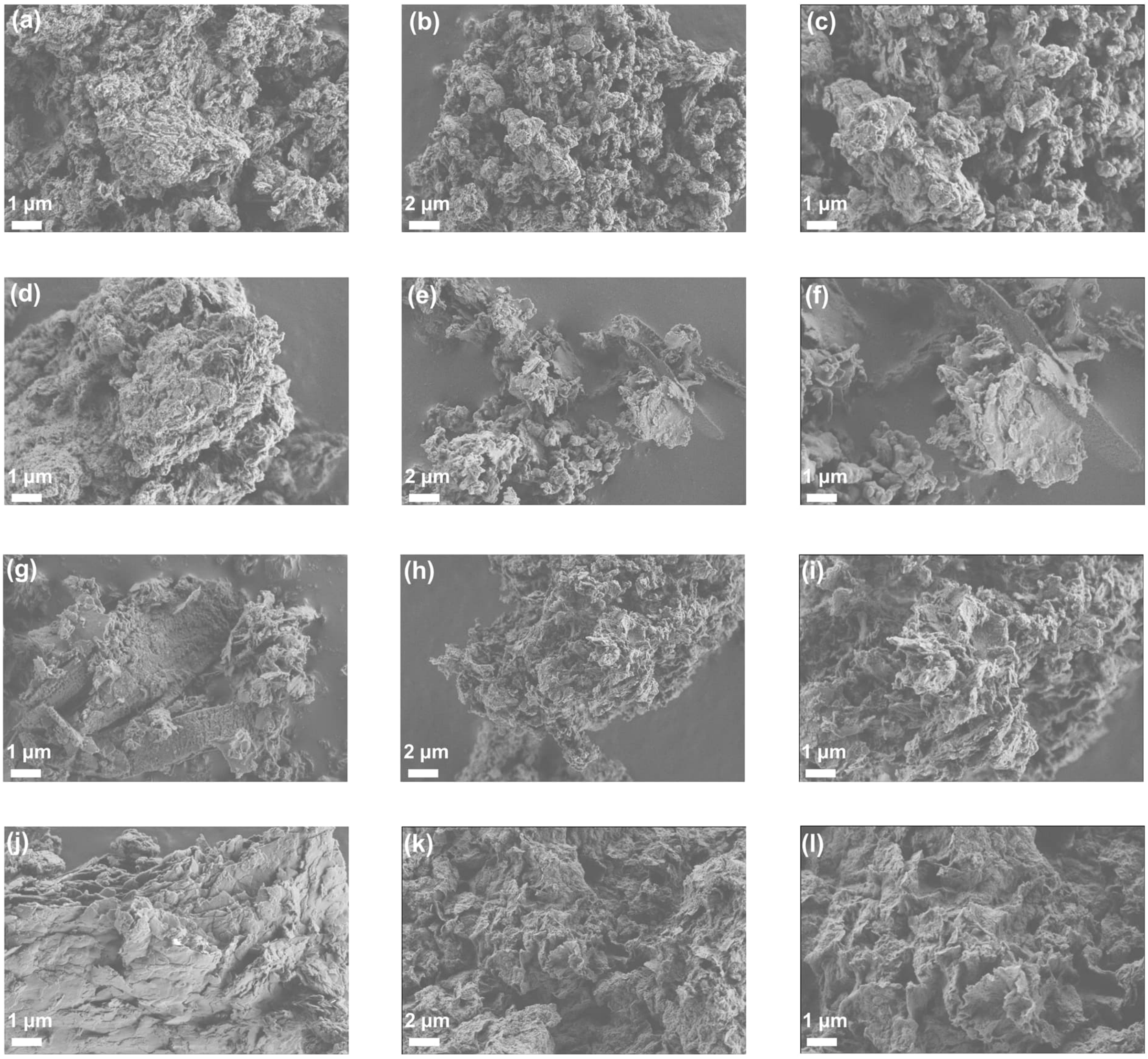

Figure S13. Scanning electron microscopy images of (a) 1D TPA-PPA polymer, (b-c) TPA-PPA/MPA POP, (c) 1D TPA-BZD polymer, (e-f) TPA-BZD/MPA POP, (g) 1D BDA-PPA polymer, (h-i) BDA-PPA/MPA POP, (j) 1D BDA-BZD polymer, and (k-l) BDA-BZD/MPA POP.

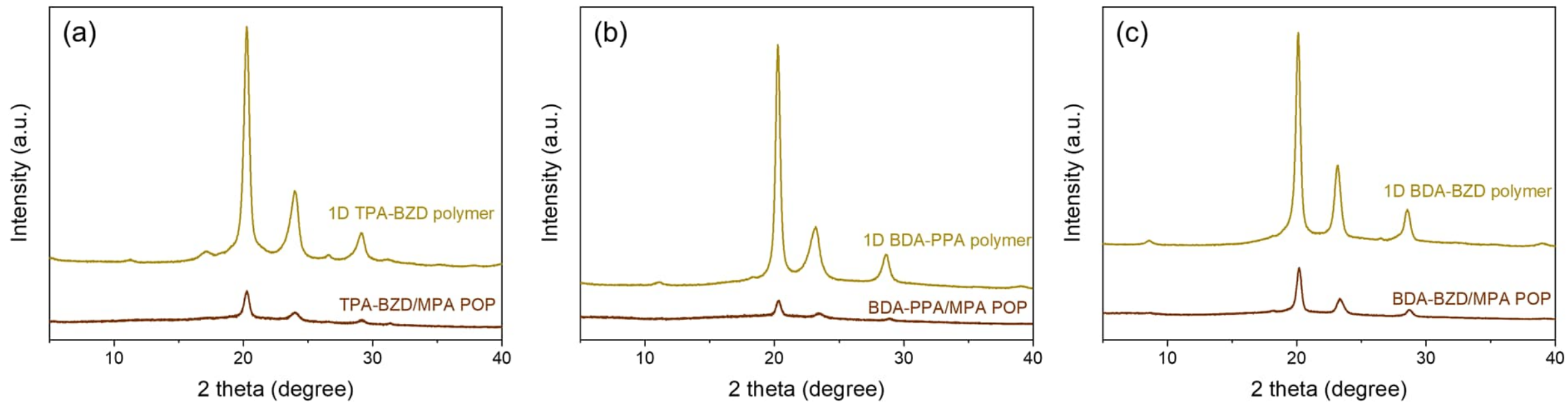

Figure S14. PXRD patterns of 1D polyimine precursors and the corresponding POPs, (a) 1D TPA-BZD polymer and TPA-BZD/MPA POP, (b) 1D BDA-PPA polymer and BDA-PPA/MPA POP, and (c) 1D BZD polymer and TPA-BZD/MPA POP.

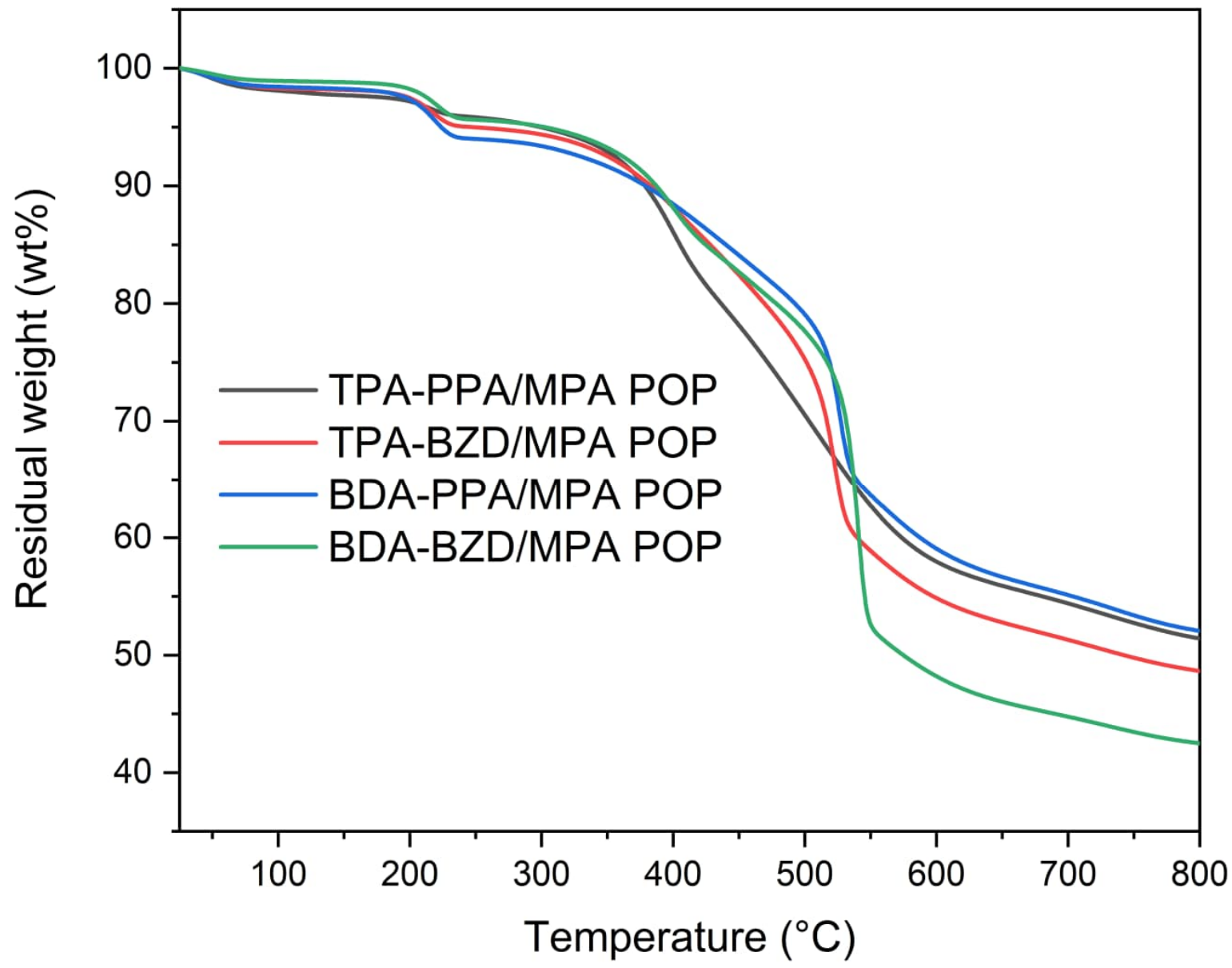

Figure S15. TGA decomposition profiles of POPs synthesized from different 1D polyimine precursors.

Further discussion on the thermogravimetric decomposition profiles of POPs

The decomposition profiles of the POPs exhibit multistep behavior, with decomposition onsets occurring at approximately 195 °C, 350 °C, and 500 °C. In comparison to the TGA profiles of their 1D polymer precursors, which generally exhibit a single decomposition event, the complex thermal degradation of the POPs can be attributed to multiple factors, including 1) the transformation of sp^2 carbons in imine linkages to sp^3 carbons in aminal linkages, leading to the formation of weaker bonds, 2) the disruption of conjugation along the polymer backbone, which can reducing stability, and 3) the presence of aminal crosslink junctions in the POP that may localize strain in the network and impact the onset of thermal degradation.[13,14] Similar multistep decomposition profiles are have been observed in aminal-linked covalent organic frameworks.[15-17]

Table S2. Calculation of the chemical costs for the synthesis of 1D TPA-PPA polymer and TPA-PPA/MPA POP[a]

Industrial scale	Chemical	Price (USD/ton)[b]	Amount (ton)	Cost (USD)
1D TPA-PPA polymer	Terephthalaldehyde (TPA)	8973	0.67	6012
	p-phenylenediamine (PPA)	10420	0.54	5627
	Ethanol	868	19.75	17149
	Glacial acetic acid	376	1.84	691
	**1D TPA-PPA polymer**	**29479**<br>**14045**[c]	1.0	29479
TPA-PPA/MPA POP	1D TPA-PPA polymer	14045	0.77	10815
	m-phenylenediamine (MPA)	3329	0.4	1332
	Trifluoroacetic acid	4342	2.56	11116
	1,4-dioxane	1447	19.2	27780
	**TPA-PPA/MPA POP**	**51043**<br>**16037**[d]	1.0	51043

Lab scale	Chemical	Price (USD/kg)[e]	Amount (kg)	Cost (USD)
1D TPA-PPA polymer	Terephthalaldehyde (TPA)	31.8	0.67	21.3
	p-phenylenediamine (PPA)	46.0	0.54	24.8
	Ethanol	2.9	19.75	57.3
	Glacial acetic acid	6.9	1.84	12.7
	**1D TPA-PPA polymer**	**116.1**	1.0	116.1
TPA-PPA/MPA POP	1D TPA-PPA polymer	116.1	0.77	89.4
	m-phenylenediamine (MPA)	37.3	0.4	14.9
	Trifluoroacetic acid	38.4	2.56	98.3
	1,4-dioxane	4.0	19.2	76.8
	**TPA-PPA/MPA POP**	**279.4**	1.0	279.4

[a] Cost calculations were based on reaction yields of 98% for the 1D TPA–PPA polymer and 85.4% for the TPA–PPA/MPA POP. Costs related to energy consumption, labor, equipment, and facility space were not included.
[b] Prices of chemicals at ton scale were obtained from https://www.100ppi.com/
[c] Solvent recycling was considered, assuming a 10 vol% loss of solvent per batch synthesis
[d] Recycling of both solvent and TFA catalyst was considered, assuming a 10 vol% loss of solvent and catalyst per batch synthesis.
[e] Prices of chemicals at laboratory scale were obtained from the supplier website www.energy-chemical.com/

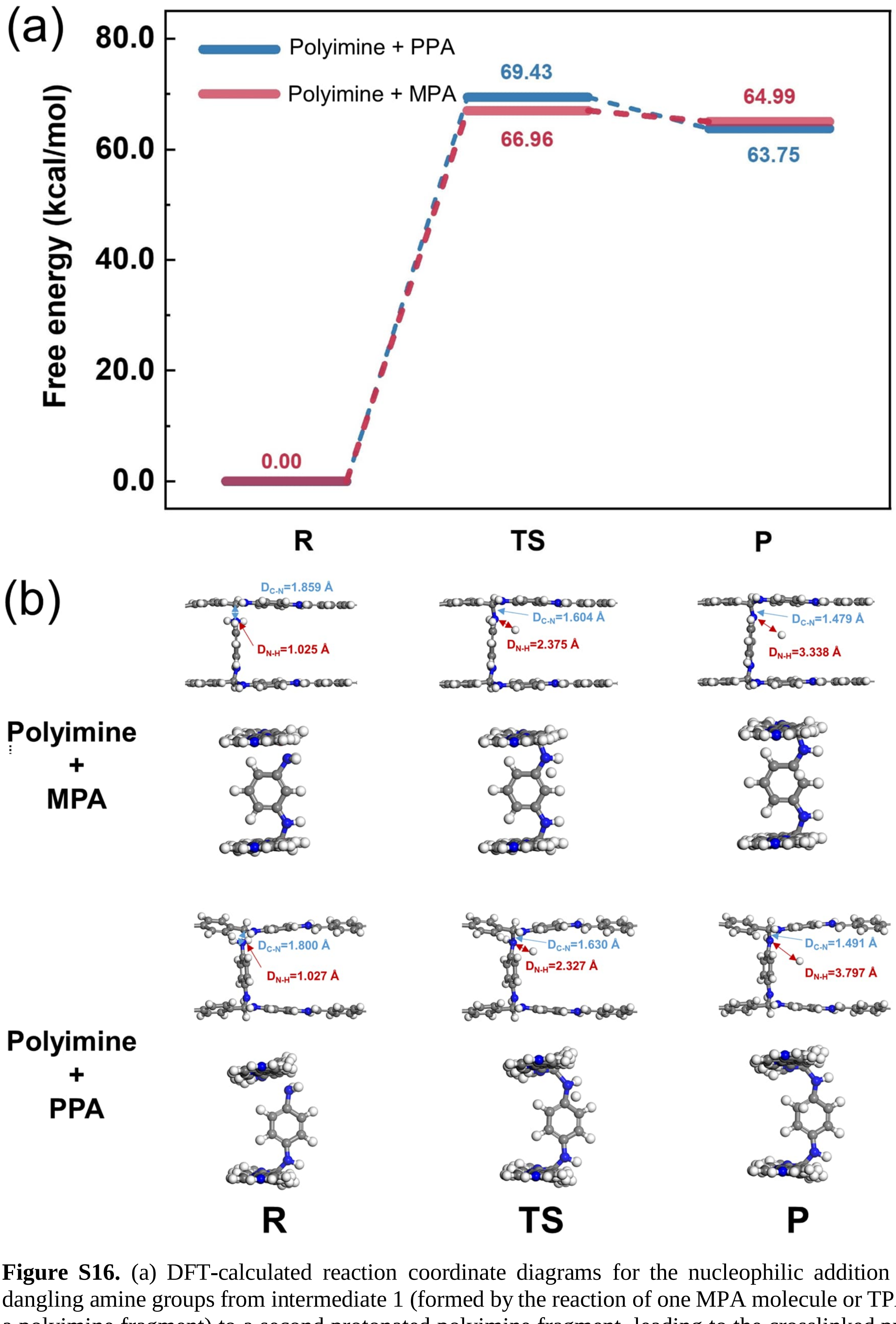

Figure S16. (a) DFT-calculated reaction coordinate diagrams for the nucleophilic addition of the dangling amine groups from intermediate 1 (formed by the reaction of one MPA molecule or TPA with a polyimine fragment) to a second protonated polyimine fragment, leading to the crosslinked product. The calculated activation free-energy barriers and relative free energies are indicated. (b) Optimized structures of the reactant, transition state, and product, shown from top and side views. Key distances are highlighted, including the C–N distance between the nucleophilic amine nitrogen and the imine carbon, and the N–H distance of the dangling amine group.

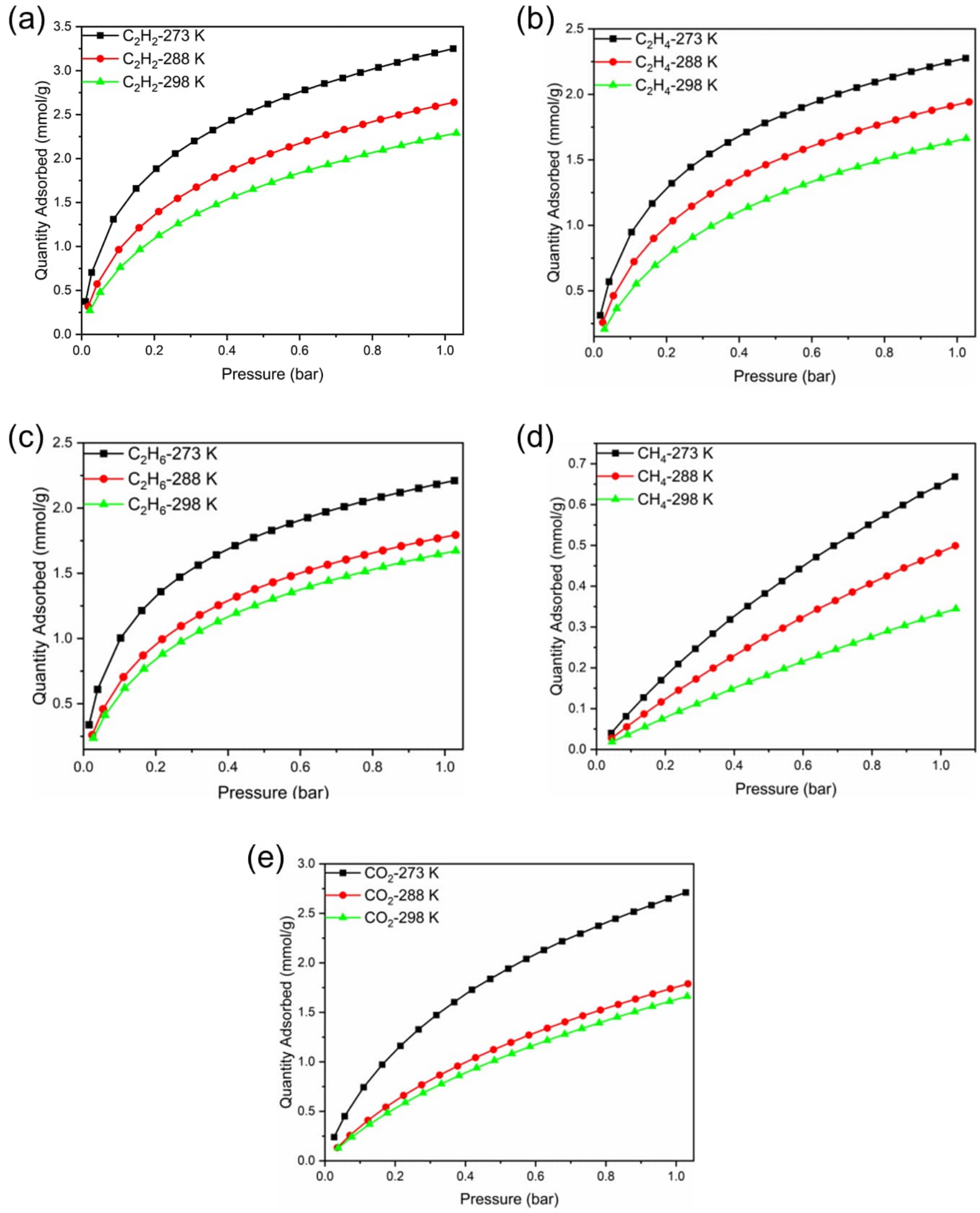

Figure S17. Adsorption isotherms of TPA–PPA/MPA POP recorded at 0 °C (273 K), 15 °C (288 K), and 25 °C (298 K) for (a) C_2H_2, (b) C_2H_4, (c) C_2H_6, (d) CH_4, and (e) CO_2.

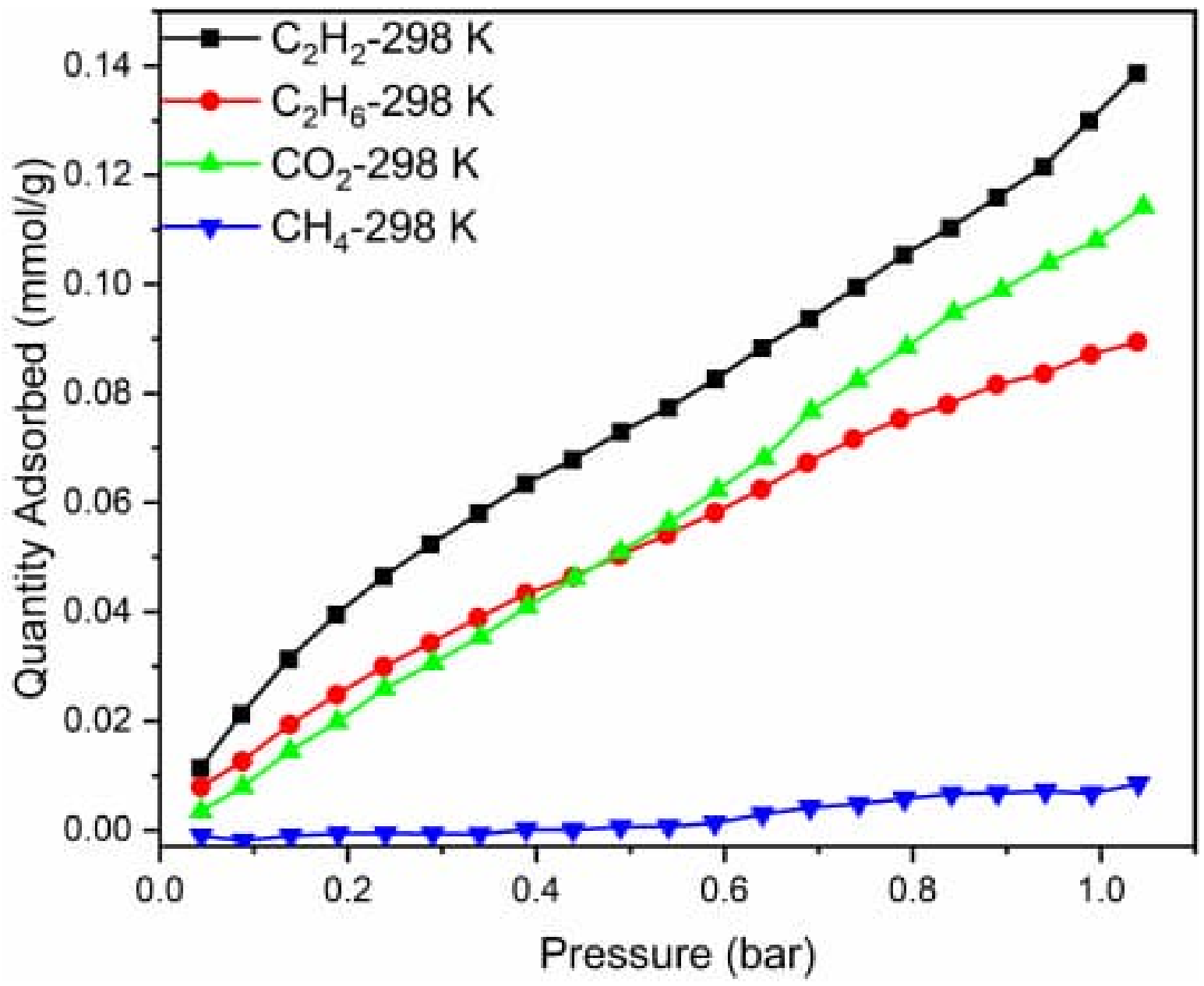

Figure S18. Adsorption isotherms of the TPA–PPA polymer for C_2H_2, C_2H_6, CH_4, and CO_2 measured at 25 °C (298 K).

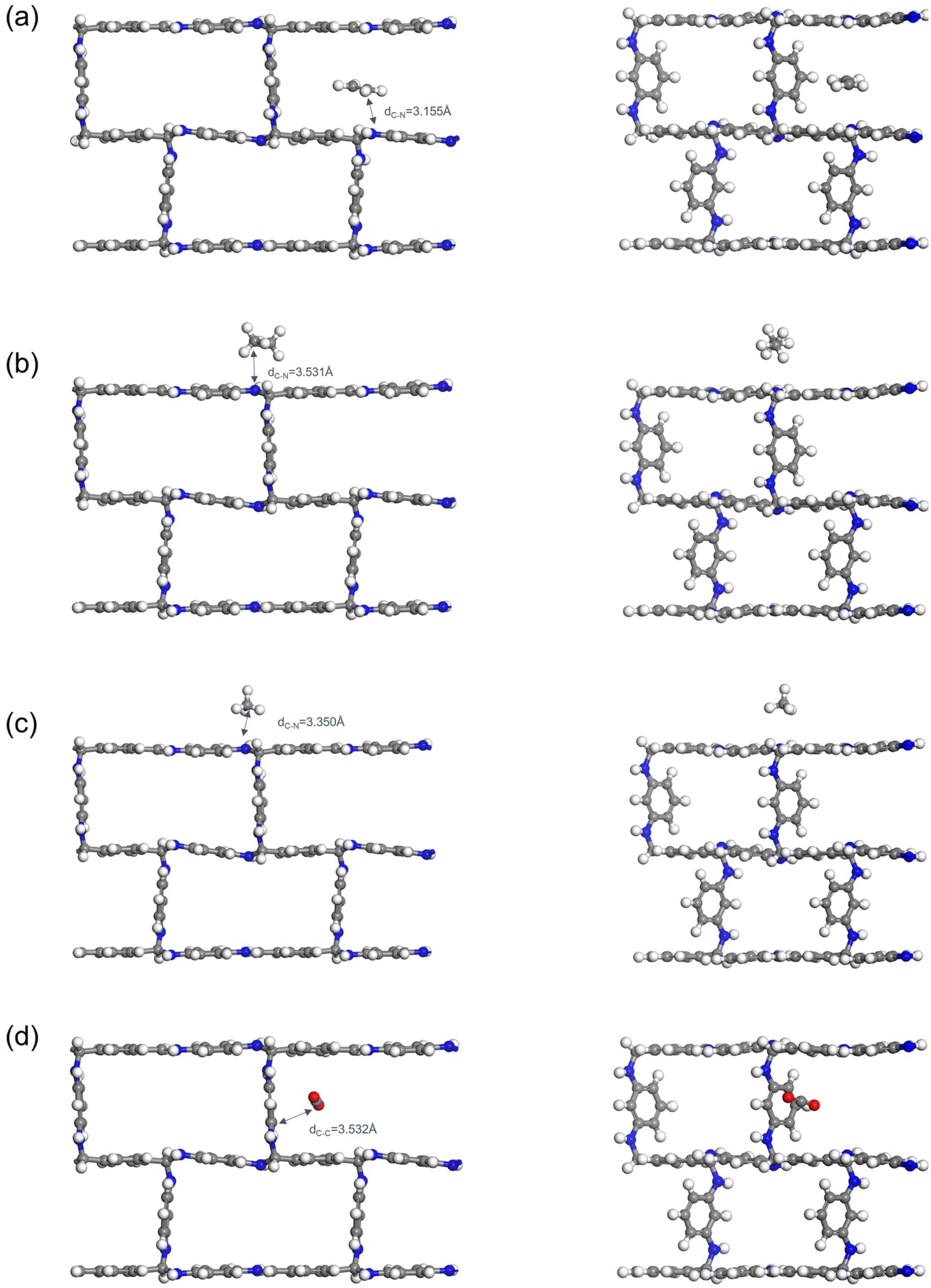

Figure S19. Optimized adsorption configurations of (a) C_2H_4, (b) C_2H_6, (c) CH_4, and (d) CO_2 on the POP, showing the distances between the TPA–PPA/MPA POP framework and the gas molecules from different views.

Table S3. Fitted parameters of gas adsorption data of TPA–PPA/MPA POP using the Langmuir model.

Gas	$q_{sat,A}$ (mmol/g)	b_A (Pa^{-1})	$q_{sat,B}$ (mmol/g)	b_B (Pa^{-1})	Adj.R^2
C_2H_2	0.87655 ± 0.04587	1.39666E-4 ± 9.04437E-6	3.2958 ± 0.04166	8.59133E-6 ± 5.48848E-7	0.99996
C_2H_4	0.62896 ± 0.0297	1.02121E-4 ± 4.95767E-6	2.27151 ± 0.01366	8.94615E-6 ± 4.13895E-7	0.99999
C_2H_6	0.7081 ± 0.03442	1.24497E-4 ± 6.67291E-6	1.95068 ± 0.01451	1.04559E-5 ± 6.06216E-7	0.99997
CO_2	0.57385 ± 0.05082	3.66721E-5 ± 2.38655E-6	5.0755 ± 0.18102	3.02339E-6 ± 2.4964E-7	0.999999
CH_4	1.84348 ± 0.01584	2.48597E-6 ± 2.54392E-8	-	-	0.999998

Note: Adsorption data were recorded at 298 K over a pressure range of 0–1 bar. The adsorption isotherms of C_2H_2, C_2H_4, C_2H_6, and CO_2 were fitted using a dual-site Langmuir model, whereas CH_4 adsorption isotherm was fitted using a single-site Langmuir model.

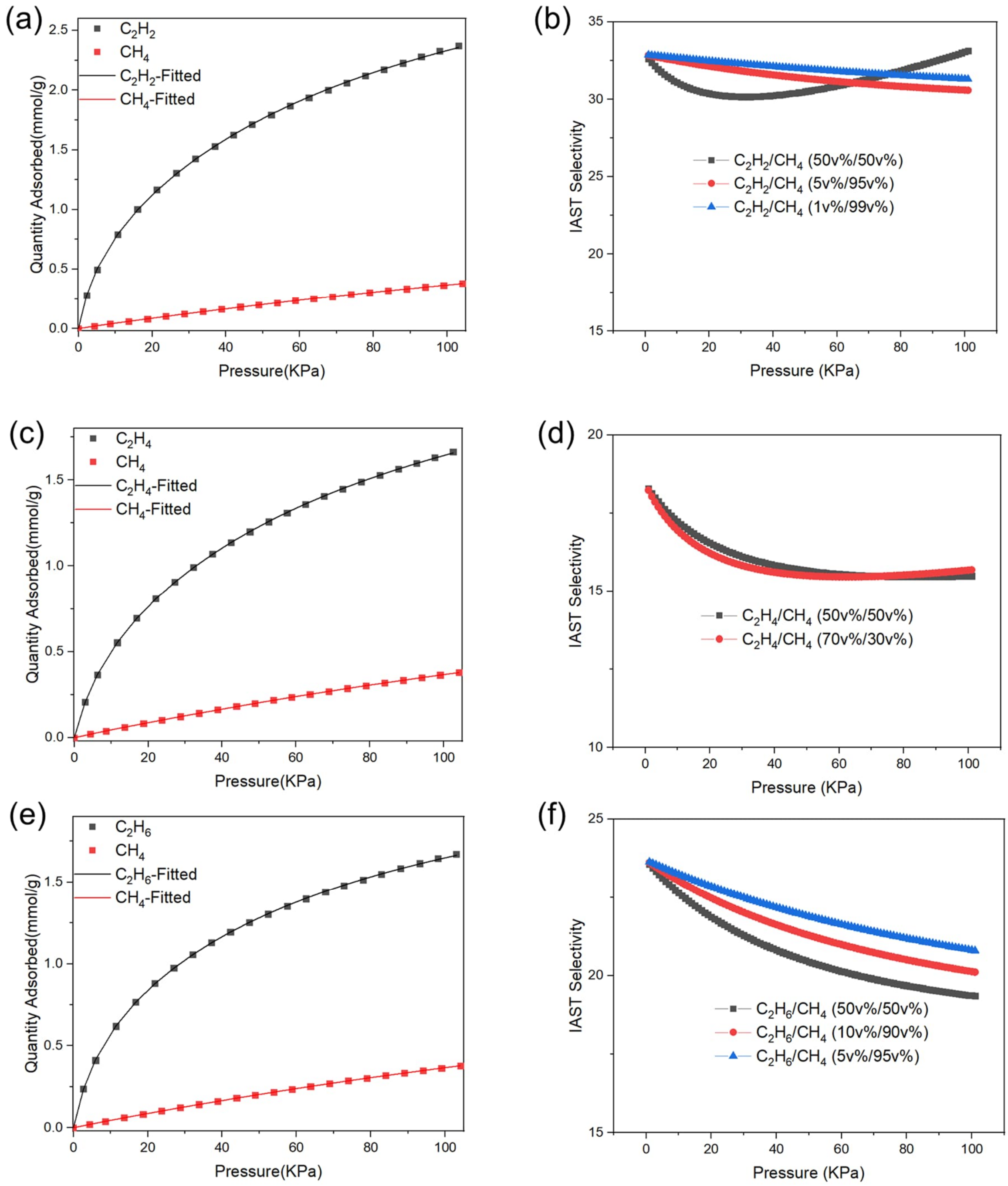

Figure S20. (a, c, e) Adsorption isotherms of C_2H_2/CH_4, C_2H_4/CH_4, and C_2H_6/CH_4 on TPA–PPA/MPA POP recorded at 25 °C (298 K), together with the corresponding fitted results. The isotherms of C_2H_2, C_2H_4, C_2H_6, and CO_2 were fitted using the dual-site Langmuir model, while CH_4 adsorption was fitted using the single-site Langmuir model. (b, d, f) IAST selectivities for C_2H_2/CH_4, C_2H_4/CH_4, and C_2H_6/CH_4 calculated from the single-component adsorption isotherms at different gas mixture ratios.

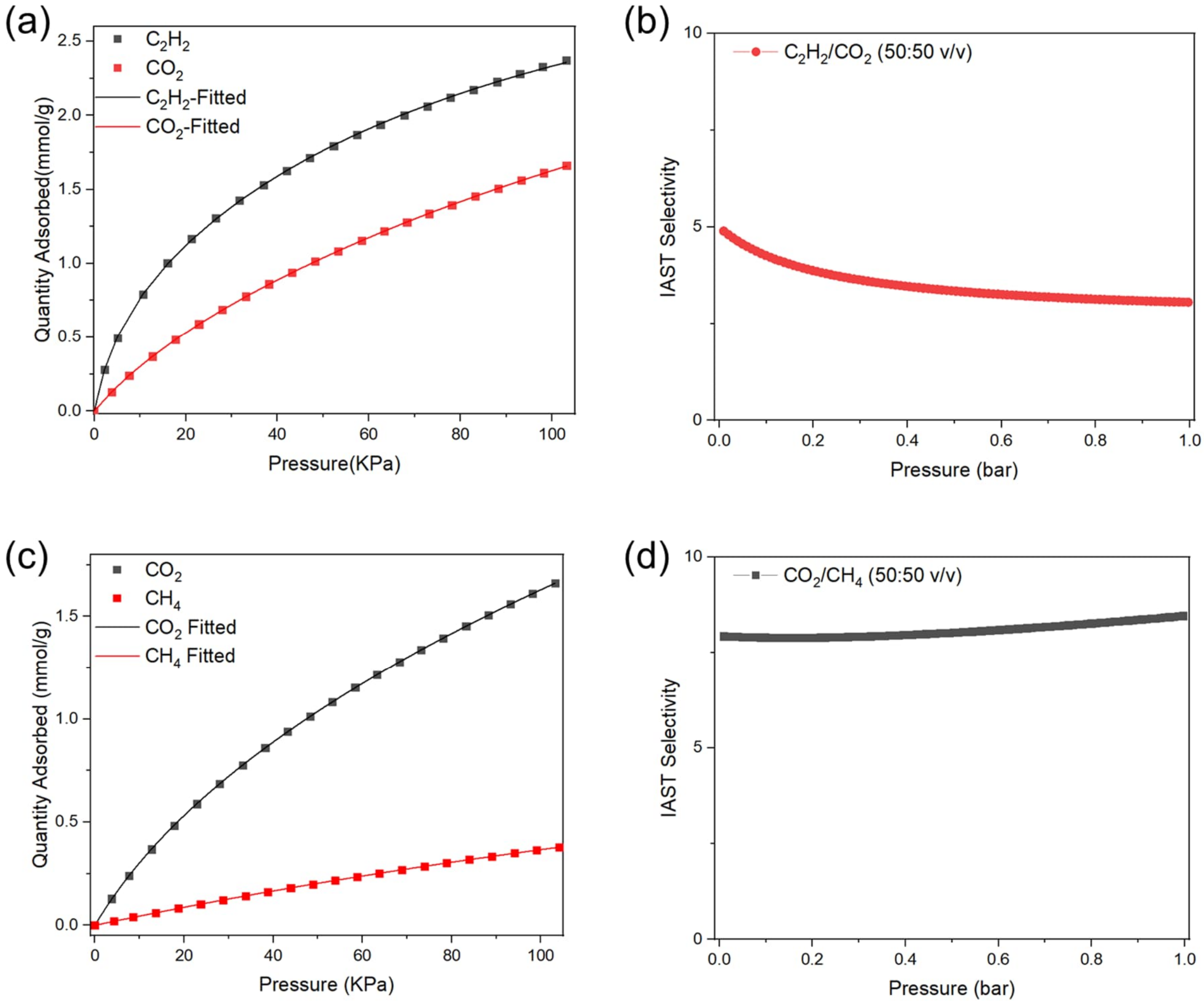

Figure S21. (a) C_2H_2 and CO_2 adsorption isotherms of TPA–PPA/MPA POP recorded at 25 °C (298 K) and the corresponding fits using the dual-Langmuir model. (b) C_2H_2/CO_2 (v/v = 50/50) selectivity calculated from the single-component adsorption isotherms using the IAST method. (c) CO_2 and CH_4 adsorption isotherms of TPA–PPA/MPA POP recorded at 25 °C (298 K) and the corresponding fits using the dual-Langmuir model. (d) CO_2/CH_4 (v/v = 50/50) selectivity calculated from the single-component adsorption isotherms using the IAST method.

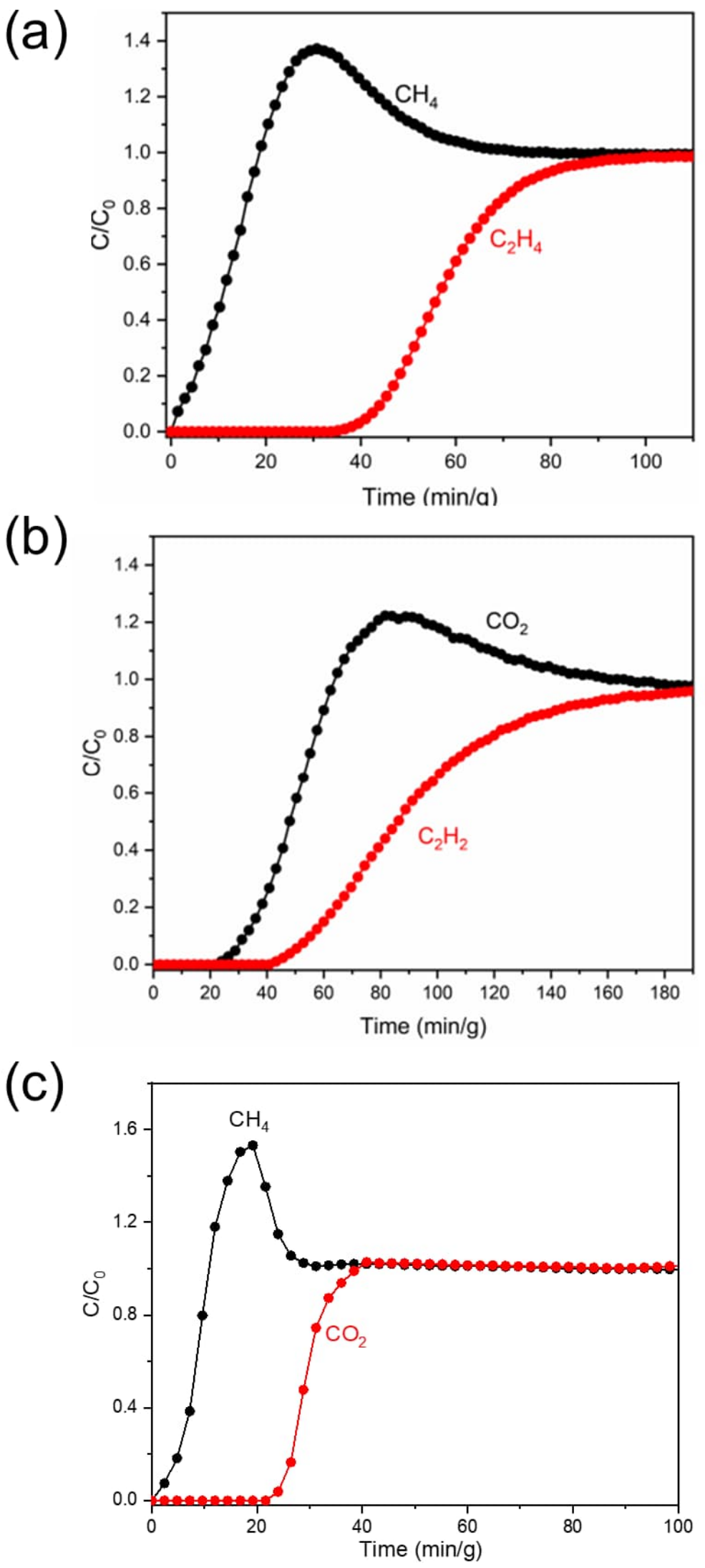

Figure S22. Dynamic breakthrough curves for the separation of (a) C_2H_4/CH_4 (70/30, v/v), (b) C_2H_2/CO_2 (50/50, v/v) and (c) CO_2/CH_4 (50/50, v/v) at 25 °C (298 K) and a flow rate of 1 mL/min, using TPA–PPA/MPA POP as a fixed-bed sorbent.

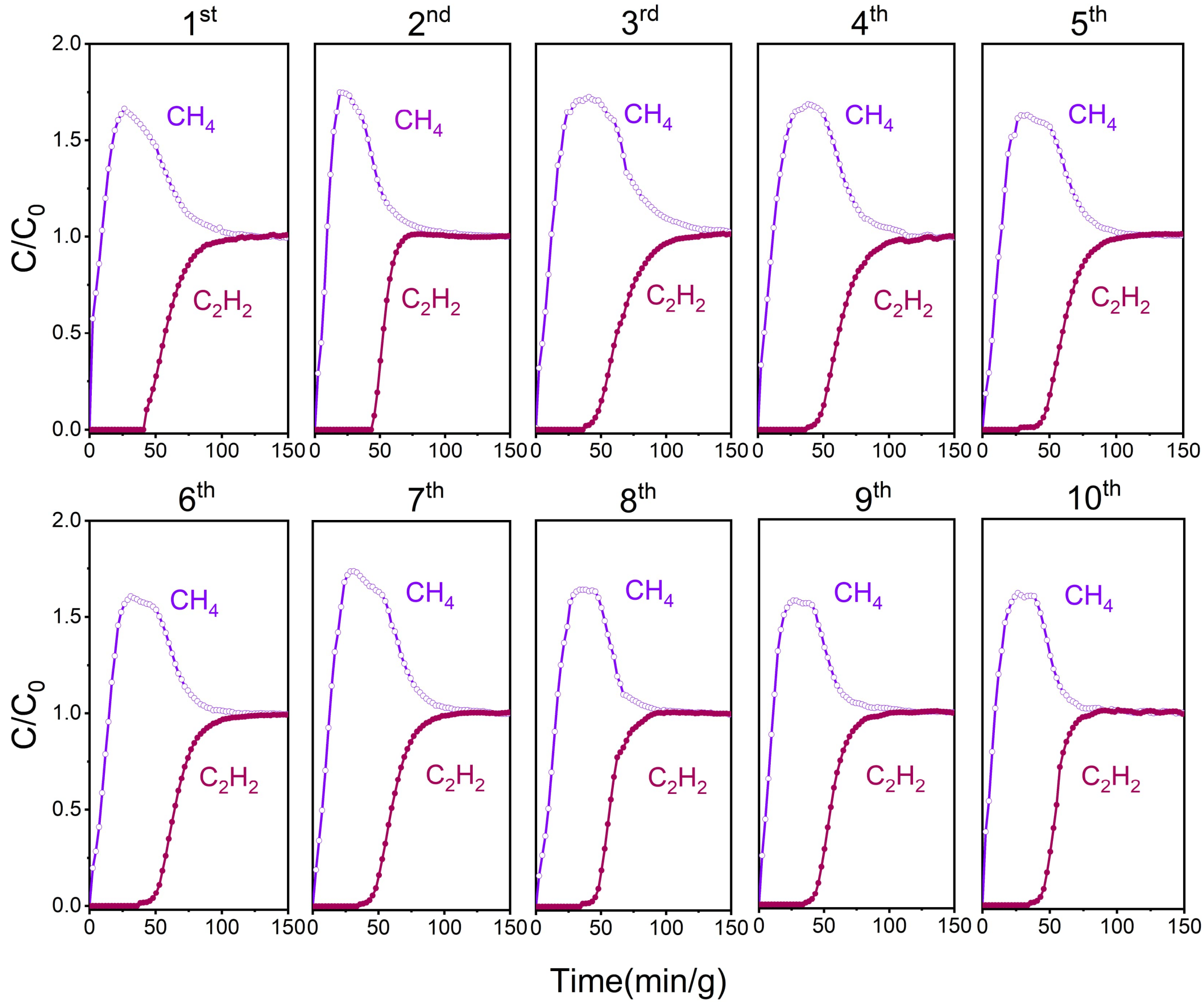

Figure S23. Dynamic breakthrough curves of large-scale synthesized TPA-PPA/MPA POP for the separation of C_2H_2/CH_4 (50/50 v/v) over 10 cycles at 25 °C (298 K) and with a gas mixture flow rate of 1 mL/min.

Table S4. Comparison of hydrocarbon and CO_2 gas uptakes and selectivities for C_2 hydrocarbon/CH_4 and C_2H_2/CO_2 separations of TPA-PPA/MPA POP with those of reported porous organic materials.

Samples	S_{BET} (m^2/g)	Gas uptake at 1 bar (mmol/g)					Selectivity[a]				Ref.
		T (K)	C_2H_2	C_2H_4	C_2H_6	CO_2	C_2H_2/ CH_4	C_2H_4/ CH_4	C_2H_6/ CH_4	C_2H_2/ CO_2	
NDA-TAPA COF	1090	273	3.9	2.52	2.44	2.56	-	-	-	-	18
		283	3.26	2.11	2.01	2.14	-	-	-	-	
		293	2.67	1.75	1.65	1.74	-	-	25.5	5.1	
TFPA-TAPA COF	1069	273	2.22	1.52	1.85	1.43	-	-	-	-	18
		283	1.81	1.28	1.45	1.15	-	-	-	-	
		293	1.44	1.12	1.07	0.91	-	-	22.5	3.5	
HOF-BTB	955	273	4.92	3.8	4.25	-	9.5	9.3	15.3	-	19
		295	2.87	2.48	3.09	-	7.8	7.9	10.8	-	
COF-Q-CF3	736	273	2.86	-	-	-	44	-	-	-	20
		298	1.96	-	-		15	-	-	-	
TT-POP-1	666	273	3.49	2.37	-	2.32	-	-	-	-	21
		298	2.23	1.63	-	1.37	88	27	-	-	
TT-POP-2	409	273	1.95	1.36	-	1.49					21
		298	1.38	0.88	-	0.91	96	31			
NKCOF-11-AA	1253	298	1.89	1.26	-	1.18	-	-	-	3.25	22
NKCOF-11-ABC	653	298	3.03	2.04	-	2.02	-	-	-	4.5	22
3D pts COF	3478	298	3.18	-	-	1.43	26	-	-	3.7	23
TP-ND	374	298	2.36	1.56	-	-	-	-	-	-	24
TP-IQD	289	298	2.05	0.71	-	-	-	-	-	-	24
TFB-IQD	101	298	0.49	0.27	-	-	-	-	-	-	24
TPA-PPA/MPA POP	548	273	3.25	2.27	2.21	2.71	-	-	-	-	This work
		288	2.64	1.94	1.79	1.79	-	-	-	-	
		298	2.37	1.66	1.67	1.66	32.6	18.3	23.4	4.9	

[a.] IAST selectivity for gas mixtures (v:v = 50%:50%);
-: Data not available

References:

(1) B. J. Delley, Chem. Phys. 1990, 92, 508-517.

(2) B. Delley, J. Chem. Phys. 2000, 113, 7756-7764.

(3) J. P. Perdew, K. Burke, M. Ernzerhof, Phys. Rev. Lett. 1996, 77, 3865-3868.

(4) L. Versluis, T. Ziegler, J. Chem. Phys. 1988, 88, 322-328.

(5) U. Barth, L. Hedin, J. Phys. C: Solid State Phys. 1972, 5, 1629.

(6) C. S. Lee, T. S. Hwang, Y. Wang, S. M. Peng, C. S. Hwang, J. Phys. Chem. 1996, 100, 2934-2941.

(7) T. Lin, W. D. Zhang, Huang, J. C. He, J. Phys. Chem. B, 2005, 109, 13755-13760.

(8) G. Kresse and J. Furthmüller, Phys. Rev. B, 1996, 54, 11169

(9) G. Kresse and D. Joubert, Phys. Rev. B, 1999, 59, 1758

(10) M. Ernzerhof and G. E. Scuseria, J. Chem. Phys., 1999, 110, 5029.

(11) K. Momma and F. Izumi, J. Appl. Crystallogr., 2011, 44, 1272.

(12) V. Wang, N. Xu, J.-C. Liu, G. Tang and W.-T. Geng, Comput. Phys. Commun., 2021, 267, 108033.

(13) C. H. Fox, G. M. ter Hurrne, R. J. Wojtecki, G. O. Jones, H. W. Horn, E. W. Meijer, C. W. Frank, J. L. Hedrick, J. M. García, Nat. Commun., 2015, 6, 7417.

(14) J. M. Winnie, L. Leibler, F. E. Du Prez, Polym. Chem., 2019, 10, 6091–6108.

(15) S.-Y. Jiang, S.-X. Gan, X. Zhang, H. Li, Q.-Y. Qi, F.-Z. Cui, J. Lu, X. Zhao, J. Am. Chem. Soc., 2019, 141, 14981-14986.

(16) J. Yang, X. Han, X. Feng, RSC Adv., 2024, 14, 21151-21157.

(17) M. Brzeziński, A. Tyszka-Gumkowska, A. Gorski, M. J. Potrzebowski, T. Polczyk, W. Wegner, S. Gawinkowski, J. Ostapko, Angew. Chem. Int. Ed. 2026, 65, e22521.

(18) A. Dey, D. Brahma, S. Laha, V. R. Bakuru, S. R. V. Parambil, A. Singh, S. Balasubramanian and T. K. Maji, Chem. Mater., 2025, 37, 4314-4324.

(19) T.-U. Yoon, S. B. Baek, D. Kim, E.-J. Kim, W.-G. Lee, B. K. Singh, M. S. Lah, Y.-S. Bae and K. S. Kim, Chem. Commun., 2018, 54, 9360-9363.

(20) Y. Wang, J. Xie, Z. Ren and Z.-H. Guan, Chem. Eng. J., 2022, 448, 137687.

(21) X. Liu, J. Du, Y. Ye, Y. Liu, S. Wang, X. Meng, X. Song, Z. Liang and W. Yan, Chin. J. Chem. Eng., 2022, 42, 64-72.

(22) Z. Wang, Y. Zhang, T. Wang, E. Lin, T. Wang, Y. Chen, P. Cheng and Z. Zhang, Small, 2023, 19, 2303684.

(23) L. Chen, C. Gong, X. Wang, F. Dai, M. Huang, X. Wu, C.-Z. Lu and Y. Peng, J. Am. Chem. Soc., 2021, 143, 10243-10249.

(24) J. Li, Z. Cheng, Z. Wang, J. Dong, H. Jiang, W. Wang, X. Zou and G. Zhu, Angew. Chem. Int. Ed., 2023, 62, e202216675.
